# Biosynthesis and Antimicrobial Activity of Pseudodesmin and Viscosinamide Cyclic Lipopeptides Produced by Pseudomonads Associated with the Cocoyam Rhizosphere

**DOI:** 10.3390/microorganisms8071079

**Published:** 2020-07-20

**Authors:** Feyisara E. Oni, Niels Geudens, Amayana Adiobo, Olumide O. Omoboye, Elsie A. Enow, Joseph T. Onyeka, Ayodeji E. Salami, René De Mot, José C. Martins, Monica Höfte

**Affiliations:** 1 Laboratory of Phytopathology, Department of Plants and Crops, Faculty of Bioscience Engineering, Ghent University, Coupure Links 653, B-9000 Ghent, Belgium; feyisaraeyiwumi.olorunleke@ugent.be (F.E.O.); oomoboye@oauife.edu.ng (O.O.O.); ayamelsie@yahoo.com (E.A.E.); 2 Unit for Environmental Sciences and Management, Faculty of Natural and Agricultural Sciences, North-West University, 2520 Potchefstroom, South Africa; 3 NMR and Structure Analysis Unit, Department of Organic and Macromolecular Chemistry, Faculty of Science, Ghent University, Krijgslaan 281, B-9000 Gent, Belgium; niels.geudens@ugent.be (N.G.); jose.martins@ugent.be (J.C.M.); 4 Jay PJ Biotechnology Laboratory, Institute of Agricultural Research for Development (IRAD), Ekona, P. M. B 25 Buea, Cameroon; amayanadio@yahoo.com; 5 Department of Microbiology, Obafemi Awolowo University, 220005 Ile-Ife, Osun State, Nigeria; 6 Plant Pathology Unit, National Root Crops Research Institute (NRCRI), 440001 Umudike, Abia State, Nigeria; jonyeka@yahoo.com; 7 Faculty of Agriculture, Department of Crop, Horticulture and Landscape Design, Ekiti State University (EKSU), 360211 Ado-Ekiti, Nigeria; ayodeji.salami@eksu.edu.ng; 8 Centre of Microbial and Plant Genetics, Faculty of Bioscience Engineering, KU Leuven, 3001 Heverlee, Belgium; rene.demot@kuleuven.be

**Keywords:** viscosin group, WLIP, evolution, CLP perception, defense response, *Pythium myriotylum*, *Rhizoctonia solani*, swarming motility

## Abstract

*Pseudomonas* cyclic lipopeptides (CLPs) are encoded non-ribosomally by biosynthetic gene clusters (BGCs) and possess diverse biological activities. In this study, we conducted chemical structure and BGC analyses with antimicrobial activity assays for two CLPs produced by *Pseudomonas* strains isolated from the cocoyam rhizosphere in Cameroon and Nigeria. LC-MS and NMR analyses showed that the *Pseudomonas* sp. COR52 and A2W4.9 produce pseudodesmin and viscosinamide, respectively. These CLPs belong to the Viscosin group characterized by a nonapeptidic moiety with a 7-membered macrocycle. Similar to other Viscosin-group CLPs, the initiatory non-ribosomal peptide synthetase (NRPS) gene of the viscosinamide BGC is situated remotely from the other two NRPS genes. In contrast, the pseudodesmin genes are all clustered in a single genomic locus. Nano- to micromolar levels of pseudodesmin and viscosinamide led to the hyphal distortion and/or disintegration of *Rhizoctonia solani* AG2-2 and *Pythium myriotylum* CMR1, whereas similar levels of White Line-Inducing Principle (WLIP), another member of the Viscosin group, resulted in complete lysis of both soil-borne phytopathogens. In addition to the identification of the biosynthetic genes of these two CLPs and the demonstration of their interaction with soil-borne pathogens, this study provides further insights regarding evolutionary divergence within the Viscosin group.

## 1. Introduction

*Pseudomonas* cyclic lipopeptides (or CLPs) are surface-active metabolites synthesized non-ribosomally by modular multifunctional enzymes named non-ribosomal peptide synthetases (NRPSs) [1]. Structurally, CLPs comprise a cyclic oligopeptide lactone ring linked to a fatty acid tail [2]. These molecules mediate multiple biological functions, including biofilm formation, swarming motility, virulence (in plant pathogenic strains), antibacterial, antifungal, anti-oomycete, insecticidal, anti-carcinogenic and antiviral properties [2,3,4,5,6,7]. These bioactive molecules are produced by specific species within the *P. syringae* complex, *P. fluorescens, P. putida* and *P. asplenii* groups [8].

*Pseudomonas* CLPs are structurally divided into 14 distinct groups [2]. From a chemical point of view, determinants of the structural variation of CLPs include overall sequence length (number of amino acids), macrocycle length and amino acid composition [2,9]. Distinct groups include the Amphisin, Bananamide, Entolysin, Orfamide, Putisolvin, Syringopeptin, Tolaasin and Xantholysin groups [2] and the more recently identified cocoyamide group [10,11]. On the other hand, genome mining enables the identification and facilitates subsequent isolation of natural products based on genetic information, even without a prior knowledge of the chemical structure [12,13]. The structural diversity of CLP condensation (C) domains enables the establishment of evolutionary relationships between closely related CLPs [14,15]. The amino acid residues that line the substrate-binding pocket in the adenylation (A) domain are predictive of the substrates that are incorporated into the growing peptide chain, thus enabling structural predictions of unknown CLPs [15]. 

The Viscosin group of CLPs comprise viscosin [16], viscosinamides [17], pseudodesmins [18], pseudophomins [19], massetolides [20] and White Line-Inducing Principle (WLIP) [21]. The members of this CLP group have an oligopeptide with nine amino acids and differ in hydrophobic amino acid identities by the presence of a Leu, Ile or Val at positions 4 and 9 and with respect to the 3-hydroxy-fatty acid tail length (10 to 12 carbons). Additional structural variations consist of either an l- or d-Leu residue at position 5 or a d-Glu or d-Gln residue at position 2. The charged CLPs possess a d-Glu2 (WLIP, viscosin, massetolide and pseudophomin), while the neutral ones possess a d-Gln2 (pseudodesmin and viscosinamide).

Within the Viscosin group, biosynthetic gene clusters (BGCs) of massetolides [22], viscosin [23], and WLIP [24,25] have been identified. However, the original pseudodesmin strain is no longer accessible and its BGC has not been described. On the other hand, the viscosinamide BGC of *P. fluorescens* DR54 is yet to be described. For characterized BGCs within the Viscosin group, the NRPS genes are part of two remote operons. It is unknown whether or not this is the norm within the Viscosin-group CLPs. 

With respect to origin, Viscosin-group CLPs such as WLIP, massetolide and viscosin have been isolated from diverse habitats including plant rhizosphere, marine organisms (tube worm and fish), frog skin, among others [11,20,26,27,28,29]. On the other hand, there have been single reports only on the isolation of the viscosinamide [17], pseudodesmin [18] and pseudophomin producers [19]. This apparent disparity in the abundance of strains producing these Viscosin-group CLPs raises the question about their functions in nature. 

Antimicrobial activities of Viscosin-group CLPs have been reported, including antibacterial activity [30,31]. In biological activity assays, purified viscosinamide produced by *P. fluorescens* DR54 reduced fungal growth and the aerial mycelium development of the plant pathogenic oomycete, *Pythium ultimum* and the basidiomycete, *R. solani* [17]. When challenged with viscosinamide, cellular changes were detected in the mycelia of both *P. ultimum* and *R. solani* [32]. Using fluorescent stains, viscosinamide-treated *P. ultimum* and *R. solani* showed extensive branching, increased hyphal septation and swelling and decreased the hydrophobicity of cell walls and membranes, among others [32]. Although several studies have been conducted using CLP members of the Viscosin group, no known study has investigated the antimicrobial activity of the structurally varied members of this group against similar plant pathogens. 

We previously described the production of at least 13 structurally diverse CLPs by *Pseudomonas* strains associated with the tropical cocoyam rhizosphere [8,11]. Preliminary Liquid Chromatography Mass Spectrometry (LC-MS) data showed that one *P. fluorescens* group strain (COR52) produces a CLP named N6, which possesses a different molecular mass (1125.42 Da) from WLIP but does not give a white line-in-agar interaction and also displays a different swarming pattern [11]. This strain was originally isolated from tissue culture-derived cocoyam plantlets grown in the disease suppressive Boteva andosol in Cameroon (unpublished data) [11]. In a preliminary study, tissue culture-derived cocoyam plantlets were grown for two weeks in an alfisol and ultisol, collected from Ado-Ekiti and Umudike, cities in Nigeria [33]. More than 90% of CLP-producing *Pseudomonas* strains isolated from these cocoyam roots showed a similar swarming phenotype, were adjudged to produce a similar CLP and clustered with the *P. fluorescens* subgroup [33]. Two of these strains were later named A2W4.9 (Ado-Ekiti) and U2W1.5 (Umudike) [34].

The tropical cocoyam crop is susceptible to the cocoyam root rot disease (CRRD), which is caused by the oomycete, *Pythium myriotylum* [11]. Given the foreknowledge that the only strain that produces N6 (COR52) was isolated from the cocoyam rhizosphere of a *Pythium* disease suppressive soil in Cameroon, whereas strains A2W4.9 and U2W1.5 were isolated from the cocoyam rhizosphere of disease conducive soils of Nigeria, we hypothesize that their corresponding CLPs likely do not show strong antimicrobial activity against *P. myriotylum* but may possess alternative functions. Thus, in our current study, we characterized the N6 CLP and putative CLPs produced by *Pseudomonas* spp. A2W4.9 and U2W1.5. Our aim was to (i) conduct chemical characterization and biosynthetic gene analysis for these putative CLPs; (ii) compare their antimicrobial activity with WLIP using two plant pathogens, namely *P. myriotylum*, and the basidiomycete, *R. solani*; (iii) to obtain insights into the relative abundance of viscosin-type CLPs.

## 2. Materials and Methods 

### 2.1. Cocoyam Tissue Culture, Plant Experiment, Bacterial Isolation and Culture Conditions

Strain and genome characteristics used in this study are listed in Table A1. Tissue culture plantlets were initiated from white cocoyam cormels collected from cocoyam fields in August 2014 at the Institute for Agricultural Research and Development (IRAD), Ekona, Cameroon. Following growth in Gamborg media [35] for eight weeks, cocoyam plantlets were transferred to the Phytopathology Laboratory, Ghent University for further multiplication. The soil collection site at Ado-Ekiti, Nigeria, was a cocoyam field located at the Ekiti State University (EKSU) Agricultural Farm while at Umudike, Nigeria, soils were collected from cocoyam experimental plots of the National Root Crops Research Institute (NRCRI). Characteristics of collected sites are detailed in [8].

Plant material was propagated by tissue culture [36] and acclimatized in potting soil (structural; Snebbout, Kaprijke, Belgium) two weeks prior to the experiment. Prior to experimental setup, approximately 100 g of air-dried soil from Ado-Ekiti and Umudike were placed in plastic pots (diameter = 15 cm) and watered till field capacity for 48 h. Subsequently, five acclimatized cocoyam plants were grown in each soil. Plants grown in Umudike soil were watered every two days, while those grown in Ado-Ekiti soil were watered once in three days in view of their different water retention capacity. Cocoyam plantlets were harvested after two weeks. Roots were washed under running tap water, blotted dry, weighed and crushed in sterile sand and 0.85% sterile saline solution mix for 2 min using sterile mortar and pestle. Thereafter, serial dilutions of 10^0^ to 10^−^^4^ were plated on King’s medium B (KB) [37] and Gould’s S1 media [38]. For colony purification, each colony was suspended in 100 µL sterile milliQ water and streaked on a KB plate for purification. This step was repeated once to ensure colony purity. Each purified colony was cultured in Luria-Bertani (LB) broth for 24 h at 150 rpm, 28 °C and stored at −80 °C. Details of all strains isolated, including A2W4.9 and U2W1.5, the enumeration of log CFU/g of roots and taxonomic analyses are elaborated elsewhere [34].

*Pseudomonas* sp. NSE1 (a WLIP producer) was isolated from the cocoyam rhizosphere at Umudike, Nigeria [33]. Strains were taken out of the −80 °C freezer when needed and streaked on King’s B media. For genomic DNA extraction, *Pseudomonas* spp. COR52, A2W4.9 and U2W1.5 were grown in 5 mL LB broth overnight at 150 rpm at 28 °C.

### 2.2. LC-MS and Nuclear Magnetic Resonance (NMR) Analysis

Seed cultures of *Pseudomonas* sp. COR52 and A2W4.9 were grown in 5 mL KB broth contained in glass tubes and placed in a rotary shaker for 24 h at 28 °C. Both strains were subsequently inoculated in 2 L flasks containing 400 mL KB broth at 150 rpm for 24 h. For each bacterial culture, ~1100 mL supernatant was collected after centrifugation at 10,000× *g* for 10 min.

Supernatants from both strains were concentrated using rotary evaporation to an approximate volume of 400 mL. Subsequently, the medium was extracted thrice using an equal volume of ethyl acetate. For pseudodesmin and viscosinamide characterization, ethyl acetate fractions were dried and the resulting precipitate was suspended in cold methyl-*tert*-butyl ether. This pre-purification step allows for enriching the crude ethyl acetate extract in its CLP content. While the CLPs do not dissolve in cold methyl-*tert*-butyl ether, many polar compounds in the extract do, allowing a purer extract to be injected onto High-performance liquid chromatography (HPLC). The resulting crude CLP fraction was then purified by repeated injections using semi-preparative HPLC. An optimal elution gradient of H_2_O/CH_3_CN was applied over 20 min at a flow rate of 17.5 mL min^−1^, while the column temperature was kept at 35 °C. All NMR measurements were performed as previously described [11].

### 2.3. Genome Sequencing, Annotation, Mining, Bioinformatics and Phylogenetic Analyses

Genomic DNA of *Pseudomonas* spp. A2W4.9, U2W1.5 and COR52 was extracted using the Wizard Genomic DNA Purification Kit (Promega Corporation, Madison, WI, USA) according to manufacturer’s instructions. Single-end or paired-end sequence reads were generated using the Illumina HiSeq2500 or MiSeq system at the BASECLEAR B. V. Leiden, Netherlands. Full details of genome sequencing and annotation are as detailed in [8].

Furthermore, genomes of previously sequenced strains were re-annotated using the Rapid Annotations Using Subsystems Technology (RAST) annotation pipeline and also submitted to antiSMASH v5.0 [39]. Details of genomes used in this study and their characteristics are listed in Table A1. Genome mining was conducted on the annotated genomes, and comparison of NRPS proteins with other protein sequences in the GenBank database was done by BLAST (Basic Local Alignment Search Tool) search. Sequences of A and C domains of the biosynthetic gene clusters (BGCs) were extracted followed by sequence alignment and phylogenetic tree construction using the Geneious 11.1.5 software (Biomatters Ltd., Auckland, New Zealand). For the in silico characterization of all CLPs, a combinational approach employing the antiSMASH v5.0 and the NRPSpredictor2 [40] enabled the prediction of the amino acid composition of the peptide moiety.

Multi-locus sequence phylogenetic analyses of CLP-producing members of the Viscosin group was conducted using housekeeping genes 16S rRNA, *rpoD*, *rpoB* and *gyrB*. These genes were extracted from the draft genome sequences of the corresponding strains. Alignments of sequences and phylogenetic tree construction were made using the Geneious 11.1.5 software. Initially, sequences of each extracted genes were aligned separately after which a concatenated sequence and tree was constructed for all four sequences. Maximum Likelihood was employed using 1000 bootstrap replicates. Multi-locus Sequence Analyses (MLSA) sequences of COR52 (Oni et al., submitted) were extracted from Genbank, while those of A2W4.9 and U2W1.5 were deposited on Genbank under the following accession numbers: U2W1.5 [MT572913 (16S rRNA), MT577352 (*rpoD*), MT577354 (*rpoB*), MT577356 (*gyrB*)) and A2W4.9 (MT572914 (16S rRNA), MT577353 (*rpoD*), MT577355 (*rpoB*), MT577357 (*gyrB*)].

### 2.4. Swarming Motility and White Line-in-Agar Assay

Swarming motility tests were conducted on 0.6% LB agar for strains used during this study, using a similar method as previously described [11]. Motility tests were also conducted on 0.6% Standard Succinate Media (SSM), which was prepared with the following components for 1 L: K_2_HPO_4_ (32.8 mM) 6 g, KH_2_PO_4_ (22 mM) 3 g, (NH_4_)_2_SO_4_ (7.6 mM) 1 g, MgSO4.7H_2_O (0.8 mM) 0.2 g, Succinate (34 mM) 9.18 g and pH adjusted to 7 with NaOH prior to autoclaving. Representative strains producing each CLP within the *P. fluorescens* group were tested for swarming motility capacity. The strains included *P. lactis* SS101 (massetolide), *P. fluorescens* SBW25 (viscosin), *Pseudomonas* sp. NSE1 (WLIP), *Pseudomonas* sp. A2W4.9 (viscosinamide), *Pseudomonas* sp. U2W1.5 (viscosinamide), *Pseudomonas* sp. COR52 (pseudodesmin).

The chemical interaction of two CLPs have been observed to yield an insoluble form of white line which surrounds the corresponding bacterial colonies. This white line-in-agar phenomenon was first observed with the co-precipitation of tolaasin with the white line-inducing principle (WLIP), a Viscosin-group CLP. Thus, white line-in-agar assays were carried out for strains producing group CLPs on Kings’ B media as described by [41]. In our previous study, *Pseudomonas* sp. CMR12a (a cocoyam rhizosphere isolate) was shown to produce phenazines and two cyclic lipopeptides namely sessilin (a tolaasin variant) and orfamide [41]. A double mutant in CMR12a which only produces sessilin interacts with an orfamide producer to give a white line. In this study, this sessilin producer (*Pseudomonas* sp. CMR12a-ΔPhz-ΔCLP2) was used as an indicator strain to test for white line-in-agar phenotype in strains producing the Viscosin-group CLPs. The *P. fluorescens* Pf-5 strain, an orfamide producer, was used as a positive control [8].

### 2.5. Antimicrobial Activity Assays

The cocoyam rhizosphere isolate, *P. myriotylum* CMR1 [42], was maintained on Potato Dextrose Agar (PDA) for three days prior to use. The bean root rot pathogen, *R. solani* AG2-2 CuHav-Rs18 [43], was maintained on the same media for four days before use. The fungal plug-CLP method was used [44]. Sterile microscopic glass slides were covered with a thin, flat layer of water agar (Bacto agar; Difco) and placed in a plastic Petri dish containing a moist sterile filter paper. An agar plug (diameter = 5 mm) taken from an actively growing colony of *R. solani* or *P. myriotylum* and placed on top at the center of each glass slide. Stock solutions of purified CLPs were made by dissolution in Dimethyl sulfoxide (DMSO). Serial dilutions of 100 nm, 1 µM, 10 µM, 25 µM and 50 µM were prepared using sterile milliQ water. For each dilution, two droplets (15 μL each) of purified CLP (WLIP, pseudodesmin or viscosinamide) were placed on two sides of the glass slide (about 2 cm from the fungal plug). For the control treatment, 0.5% DMSO was used. All plates were incubated for 48 h (*P. myriotylum*) or 72 h (*R. solani*) at 28 °C before evaluation under an Olympus BX51 microscope. The images were processed using TopView software. Using a vernier caliper, the inhibition zones were measured as the distance between the CLP droplet and the growing edge of the pathogen. The growth inhibition effect of pure compounds towards both plant pathogens was expressed relative to the mycelial growth in the control. Five replicates of each slide were used. Representative photos are shown. 

For pathogen perception experiments, the afore-described plug-CLP droplet method was employed with slight modifications. Only one 15 µL CLP droplet was introduced 2 cm away from the fungal plug, while the other side of the plug remained blank. Results were assessed by observing whether or not the pathogen only grew towards the CLP droplet or in both directions. Pathogen-CLP interaction was evaluated microscopically and pictures were taken.

## 3. Results

### 3.1. Chemical Structural Elucidation of Isolated CLPs

**Figure 1:**
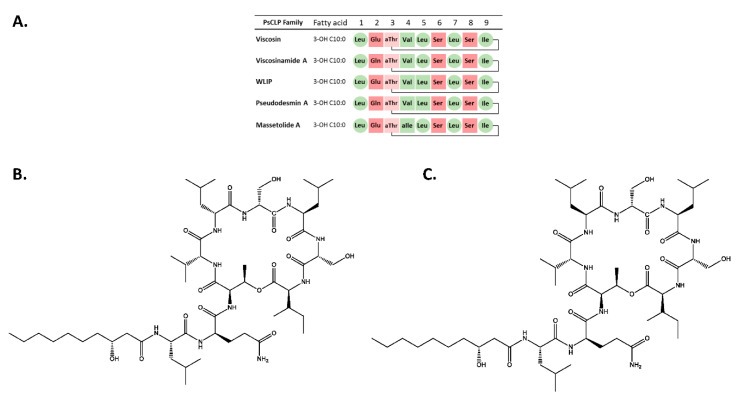
Chemical structure of pseudodesmin and viscosinamide. (**A**) Sequence comparison with other cyclic lipopeptides (CLPs) of the Viscosin group; PsCLP: *Pseudomonas* CLP. (**B**) Chemical structure of the major isolated CLP of *Pseudomonas* sp. COR52, identified as pseudodesmin A; (**C**) Chemical structure of the major isolated CLP of *Pseudomonas* sp. A2W4.9, identified as viscosinamide A. Hydrophobic amino acids are indicated in green, hydrophilic amino acids are indicated in pink. d-amino acids are square-shaped, while l-amino acids are circled.

**Figure A1:**
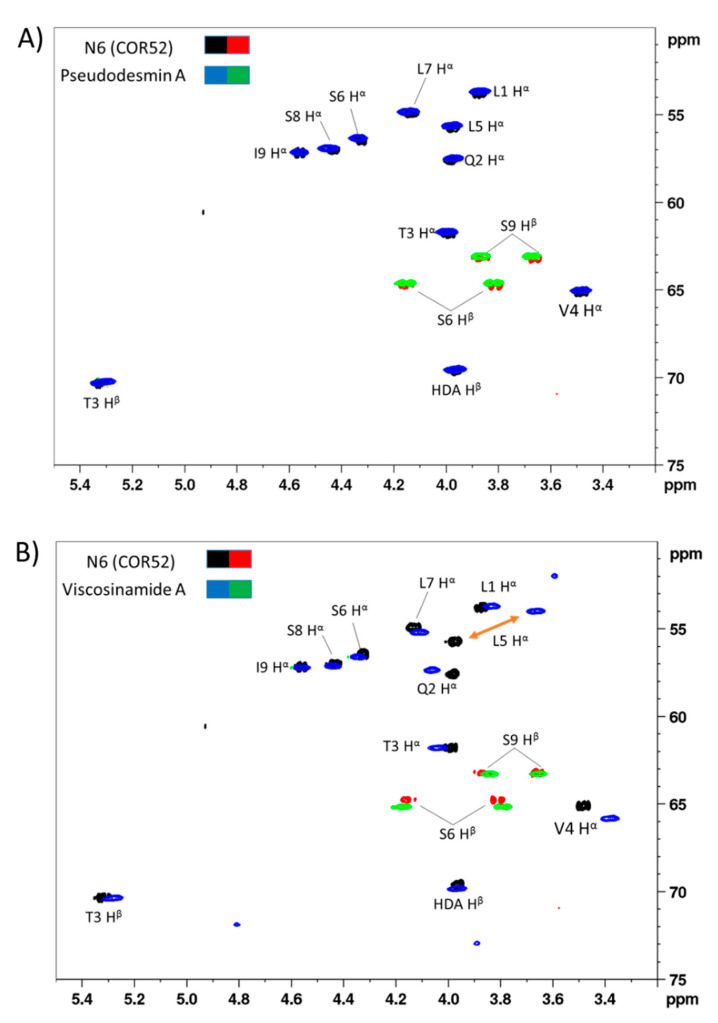
(**A**) Overlay of the alpha region of a ^1^H-^13^C gHSQC spectrum of the isolated CLP (black/red) with that of pseudodesmin A (blue/green) recorded under identical conditions. Both spectra are undistinguishable, showing that N6 is identical to pseudodesmin A. (**B**) Overlay of the alpha region of a ^1^H-^13^C gHSQC spectrum of the isolated CLP (black/red) with that of viscosinamide A (blue/green) recorded under identical conditions. A significant difference occurs for the ^1^H and ^13^C chemical shifts of Leu5. Likely, N6 has a different stereochemistry at this position.

For the CLP extracted from *Pseudomonas* sp. COR52, a 2D ^1^H-^1^H TOCSY revealed the presence of a 3-hydroxy fatty acid linked to a peptide chain involving nine amino acids (3× Leu, 2× Ser, 1× Ile, 1× Gln, 1× Val and 1× Thr) (Figure 1A,B). The amino acid sequence was confirmed by a 2D ^1^H–^1^H ROESY analysis and is detailed in Table S1. The position of the ester bond that cyclizes the molecule could be confirmed by analysis of the ^1^H–^13^C gHMBC spectrum, showing a clear cross peak between Thr3 H^β^ and Ile9 C’. Moreover, the unusually high chemical shifts of Thr3 H^β^ indicates that this residue is involved in the depsi bond. In this way, it was established that the main CLP produced by Pseudomonas sp. COR52—initially named N6—is a member of the viscosin (9:7) group. More specifically, the amino acid sequence is identical to that of viscosinamide A and pseudodesmin A. Both compounds feature the same amino acid sequence, since they only differ from one another by the presence of l-Leu5 and d-Leu5, respectively. By means of NMR spectroscopy, it could be established that the stereochemistry of the isolated CLP is identical to that of pseudodesmin A (Figure 1A,B, Figure A1, Figure S1A,B, Table S1). In short, the identity of a compound with unknown stereochemistry may be obtained relative to that of a reference compound by matching their NMR spectra recorded under identical conditions (Figure A1).

**Figure A2:**
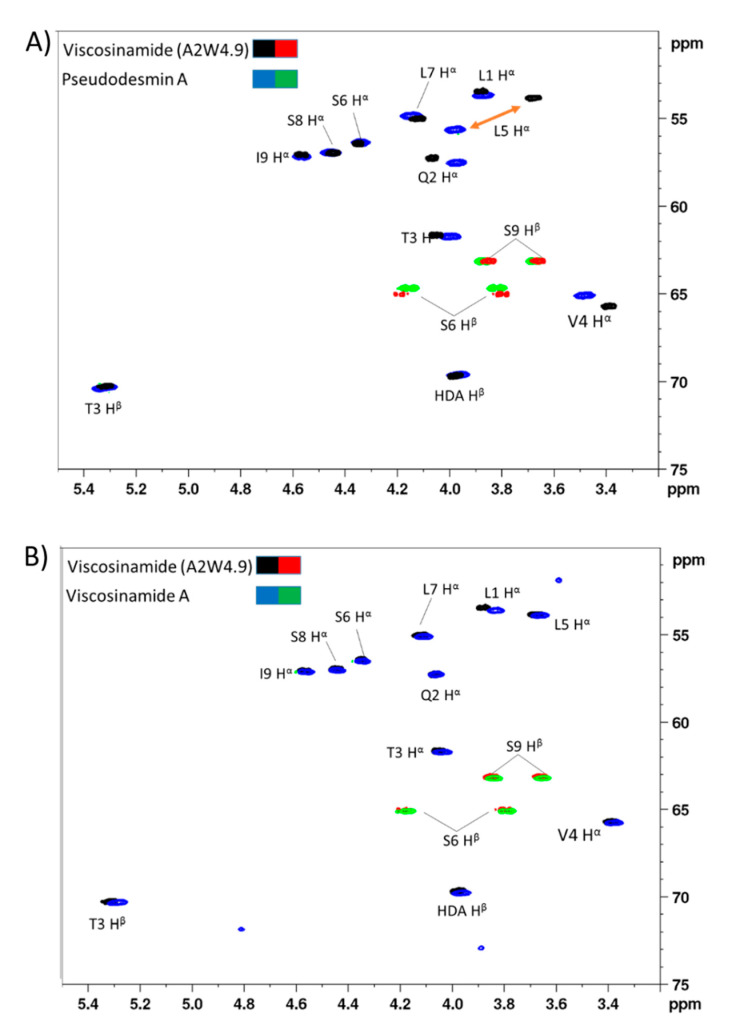
(**A**) Overlay of the alpha region of a ^1^H-^13^C gHSQC spectrum of the isolated CLP (black/red) with that of pseudodesmin A (blue/green) recorded under identical conditions. A significant difference occurs for the ^1^H and ^13^C chemical shifts of Leu5. It is likely that the isolated CLP has a different stereochemistry at this position. (**B**) Overlay of the alpha region of a ^1^H-^13^C gHSQC spectrum of the isolated CLP (black/red) with that of viscosinamide A (blue/green) recorded under identical conditions. Both spectra are undistinguishable, showing that the isolated CLP of *Pseudomonas* sp. A2W4.9 is identical to viscosinamide A.

Using a similar approach, it was found that the CLP extracted from *Pseudomonas* sp. A2W4.9 consists of a 3-hydroxy fatty acid linked to a peptide chain involving nine amino acids (3× Leu, 2× Ser, 1× Ile, 1× Gln, 1× Val and 1× Thr) (Figure 1A or Figure 1C, Figure S2A,B). The amino acid sequence was confirmed by 2D ^1^H–^1^H ROESY analysis and found to be identical to that found for the CLP extracted from Pseudomonas sp. COR52 (3-OH C10:0–Leu1–Gln2–Thr3–Val4–Leu5–Ser6–Leu7–Ser8–Ile9) (Table S2). The position of the ester bond that cyclizes the molecule could not be confirmed by analysis of the ^1^H-^13^C gHMBC spectrum due to low signal-to-noise. However, the unusually high chemical shifts of Thr3 H^β^ indicates that this residue is involved in the depsi bond. We established that the main CLP produced by *Pseudomonas* sp. A2W4.9 is a member of the Viscosin group and that the amino acid sequence is an identical match with that of viscosinamide A and pseudodesmin A. By comparison of the NMR spectra, the stereochemistry of the isolated CLP was compared to that of these two reference compounds. In this way, the isolated CLP could be unequivocally identified as viscosinamide A, including its stereochemistry (Figure A2).

### 3.2. In Silico Analysis of the Pseudodesmin and Viscosinamide Biosynthetic Gene Cluster

**Figure 2:**
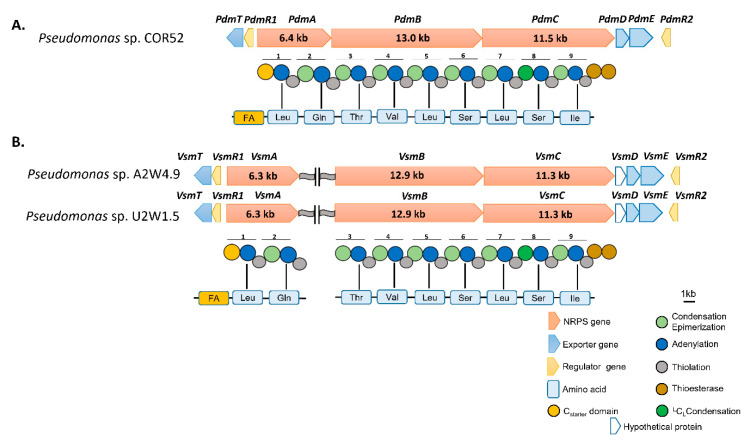
Organization of the pseudodesmin and viscosinamide biosynthetic gene clusters (BGCs). (**A**) The pseudodesmin gene cluster of *Pseudomonas* sp. COR52 comprises three non-ribosomal peptide synthetase (NRPS) genes, *pdmA*, *pdmB* and *pdmC*, in an operon-like organization. (**B**) The viscosinamide gene clusters of *Pseudomonas* spp. A2W4.9 and U2W1.5 comprise three NRPS genes, of which the initiatory NRPS gene, *vsmA*, is located distantly from the other two NRPS genes, *vsmB* and *vsmC*. The predicted peptide sequence is based on the substrate specificity of the nine modules that were derived from the *in silico* and phylogenetic analysis of the corresponding A domain sequences. FA: fatty acid.

**Figure 3:**
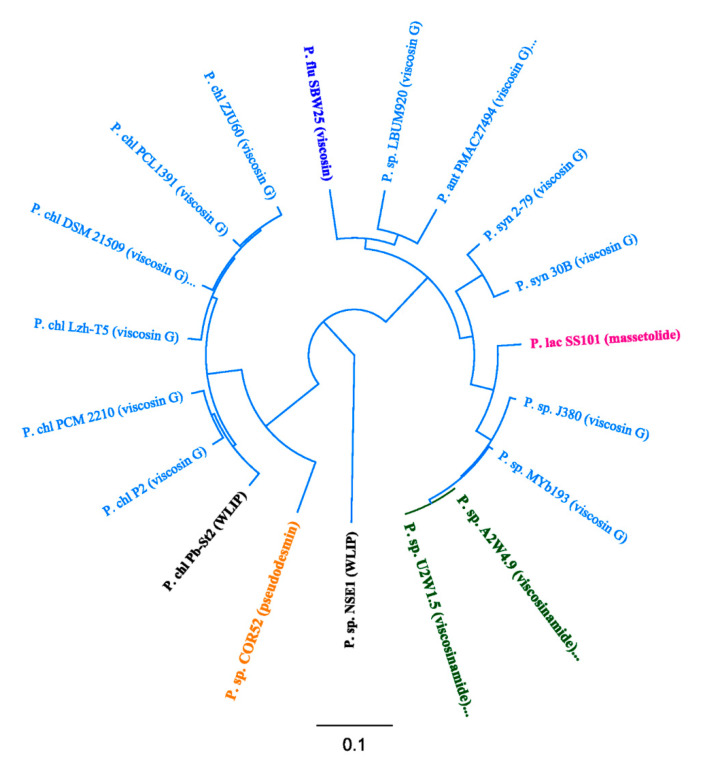
Phylogenetic analyses of the non-ribosomal peptide synthetases (NRPSs) encoded by pseudodesmin and viscosinamide BGCs strains and by selected BGCs of the Viscosin group. The cladogram of a neighbor joining tree was inferred from the alignment of collinearly concatenated NRPS sequences for producers of pseudodesmin (*Pseudomonas* sp. COR52), viscosinamide (*Pseudomonas* sp. A2W4.9, *Pseudomonas* sp. U2W1.5), White Line-Inducing Principle (WLIP) (*Pseudomonas* sp. NSE1, *P. chlororaphis* Pb-St2), massetolide (*P. lactis* SS101) and viscosin (*P. fluorescens* SBW25) and select putative producers of Viscosin-group (viscosin G) CLPs (*Pseudomonas* sp. Myb193, *Pseudomonas* sp. J380, *P. synxantha* 30B, *P. synxantha* 2-79, *P. antarctica* PAMC 27494, *Pseudomonas* sp. LBUM920, *P. chlororaphis* subsp. *piscium* PCL1391, *P. chlororaphis* subsp. *aurantiaca* DSM 19603^T^, *P. chlororaphis* subsp. *aurantiaca* PCM 2210, *P. chlororaphis* subsp. *aureofaciens* P2, P. *chlororaphis* subsp. *piscium* DSM 21509, *P. chlororaphis* subsp. *piscium* ZJU60 and *P. chlororaphis* Lzh-T5). The scale bar represents 0.1 substitutions per site. Abbreviations for species: *P. ant*, *P. antarctica*; *P. chl*, *P. chlororaphis*; *P. flu*, *P. fluorescens*; *P. lac*, *P. lactis*; *P. syn*, *P. synxantha*; *P*. sp., *Pseudomonas* sp.

For pseudodesmin gene cluster characterization, the draft genome of COR52 (Boteva, Cameroon) was analysed via antiSMASH v5.0, which revealed a single NRPS cluster comprising three genes: *pdmA* (two modules, 6.4 kb), *pdmB* (four modules, 13.0 kb) and *pdmC* (three modules, 11.5 kb) (Figure 2A). Each module predicted an amino acid and bioinformatic analyses revealed the sequence for nine modules: Leu1–Gln2–Thr3–Val4–Leu5–Ser6–Leu7–Ser8–Ile9. Similar to the BGCs of most CLP-producing strains, the *pdmA* gene is flanked upstream by a *luxR*-family regulatory gene (*pdmR1*) and a *nodT*-like transporter gene (*pdmT*), whereas the *pdmC* gene is flanked downstream by the additional transporter genes related to *macA* (*pdmD*) and *macB* (*pdmE*), as well as a second *luxR*-like gene (*pdmR2*). Phylogenetic analyses of the pseudodesmin BGC protein sequences and those belonging to the Viscosin group showed their clustering with the WLIP BGC of *P. chlororaphis* Pb-St2 and putative Viscosin group BGCs of *P. chlororaphis* strains, including P2, PCM 2210 and DSM 21,509 (Figure 3).

By similar analysis, the draft genomes of strains A2W4.9 (Ado-Ekiti, Nigeria) and U2W1.5 (Umudike, Nigeria) revealed two separate NRPS clusters comprising two and seven modules. The first cluster contains *vsmA* (two modules, 6.3 kb) and the second carries *vsmB* (four modules, 12.9 kb) and *vsmC* (three modules, 11.3 kb) (Figure 2B). For each genome, each module predicted an amino acid and bioinformatic analyses revealed the sequence for nine modules: Leu1 – Gln2 – Thr3 – Val4 – Leu5 – Ser6 – Leu7 - Ser8 – Ile9. Similar to the BGCs of known Viscosin group members, the *vsmA* gene is flanked upstream by *luxR*-like (*vsmR1*) and *nodT*-like (*vsmT*) genes, whereas the *vsmC* gene is flanked downstream by *macA*- (*vsmD*), *macB*- (*vsmE*) and *luxR*-like (*vsmR2*) genes. Similar phylogenetic analyses of the viscosinamide NRPSs showed the exact identity of both viscosinamide enzyme systems (Figure 3). These NRPS systems are nearly identical (99.2%) to those of viscosinamide producer *P. fluorescens* DR54 (L. Girard and R. De Mot, unpublished). Furthermore, these NRPSs are closely related to the undescribed ones identified in the genome of a few other strains, such as Myb193 and J380 (hence, putative viscosinamide producers). Based on NRPS comparisons, the closest known non-viscosinamide BGC is the one of massetolide producer *P. lactis* SS101 (Figure 3).

**Figure 4:**
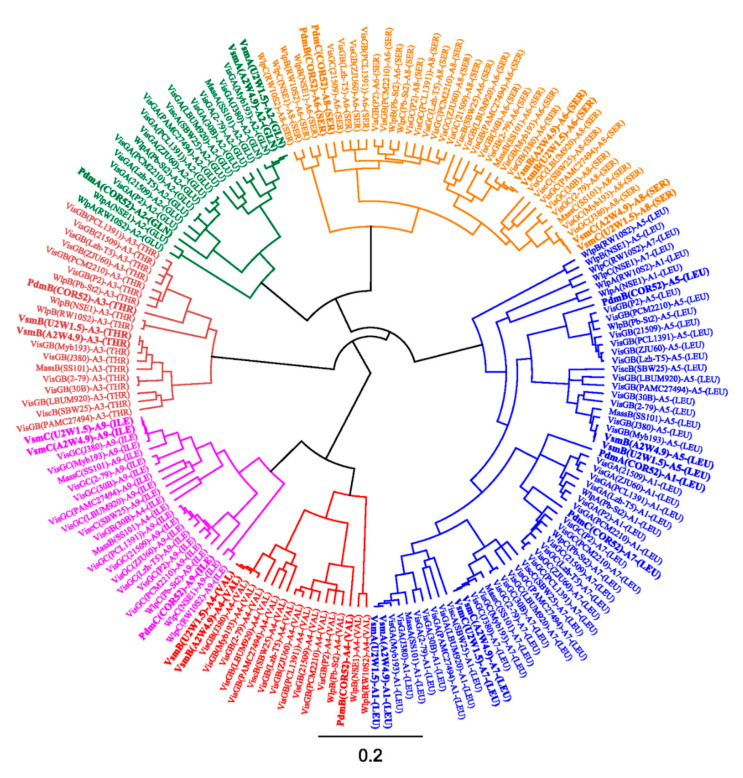
Phylogeny-based substrate prediction of pseudodesmin and viscosinamide synthetases. Cladogram of Maximum likelihood tree inferred from amino acid sequence alignment of adenylation (A) domains extracted from functionally characterized and select putative *Pseudomonas* NRPSs. Lipopeptide-specific codes used for NRPS enzymes: Pdm (pseudodesmin, *Pseudomonas* sp. COR52, highlighted in bold font); Vsm (viscosinamide, *Pseudomonas* sp. A2W4.9 and *Pseudomonas* sp. U2W1.5, highlighted in bold font); Wlp (white line inducing principle, *Pseudomonas* sp. NSE1, *P. putida* RW10S2, *P. chlororaphis* Pb-St2); Mass (massetolide, *P. lactis* SS101); Visc (viscosin, *P. fluorescens* SBW25); VisG (Viscosin group, not characterized: *Pseudomonas* sp. Myb193, *Pseudomonas* sp. J380, *P. synxantha* 30B, *P. synxantha* 2-79, *P. antarctica* PAMC 27494, *Pseudomonas* sp. LBUM920, *P. chlororaphis* subsp. *piscium* PCL1391, *P. chlororaphis* subsp. *aurantiaca* DSM 19603T, *P. chlororaphis* subsp. *aurantiaca* PCM 2210, *P. chlororaphis* subsp. *aureofaciens* P2, *P. chlororaphis* subsp. *piscium* DSM 21509, *P. chlororaphis* subsp. *piscium* ZJU60 and *P. chlororaphis* Lzh-T5). For each domain, the substrate specificity is indicated in parentheses, using the standard amino acid three-letter code. VisGA, VisGB, VisGC represent NRPS enzymes for viscosin-group CLPs that have been characterized based on genome mining only. A phylogenetic tree was drawn using the Geneious 11.1.5 software (Biomatters Ltd., Auckland, New Zealand).

Phylogenetic analyses of the A-domain substrate specificity of the nine modules enabled prediction of the peptide sequences (Figure 4). A-domains extracted from viscosinamide BGCs are closely related to those of putative viscosinamide producers Myb193 and J380 while those of the pseudodesmin BGCs occupied distinct branches in clusters with the A-domains retrieved from the WLIP producer Pb-St2 and other *P. chlororaphis* strains. The predictions are largely consistent with the peptide sequence of viscosinamide (produced by A2W4.9 and U2W1.5) and pseudodesmin (COR52) characterized by NMR. However, for both CLPs, the phylogenetic approach cannot discriminate between the incorporation of Glu or Gln in the second position.

**Figure A3:**
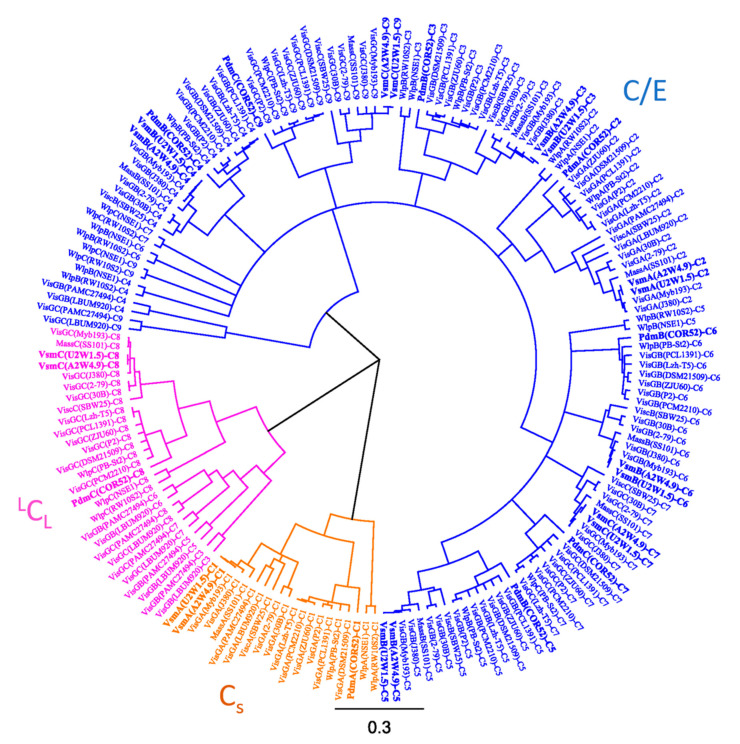
Phylogeny-based analyses of 180 condensation (C) domains extracted from NRPSs belonging to the Viscosin group. Cladogram of a maximum likelihood tree inferred from the amino acid sequence alignment of C domains extracted from functionally characterized and select putative *Pseudomonas* NRPSs. Lipopeptide-specific codes used for NRPS enzymes: Pdm (pseudodesmin, *Pseudomonas* sp. COR52, highlighted in bold font); Vsm (viscosinamide, *Pseudomonas* sp. A2W4.9 and *Pseudomonas* sp. U2W1.5, highlighted in bold font); Wlp (white line inducing principle, *Pseudomonas* sp. NSE1, *P. putida* RW10S2, *P. chlororaphis* Pb-St2); Mass (massetolide, *P. lactis* SS101); Visc (viscosin, *P. fluorescens* SBW25); VisG (Viscosin group, not characterized: *Pseudomonas* sp. Myb193, *Pseudomonas* sp. J380, *P. synxantha* 30B, *P. synxantha* 2-79, *P. antarctica* PAMC 27494, *Pseudomonas* sp. LBUM920, *P. chlororaphis* subsp. *piscium* PCL1391, *P. chlororaphis* subsp. *aurantiaca* PCM 2210, *P. chlororaphis* subsp. *aureofaciens* P2, *P. chlororaphis* subsp. *piscium* DSM 21509, *P. chlororaphis* subsp. *piscium* ZJU60 and *P. chlororaphis* Lzh-T5). For each domain, the substrate specificity is indicated in parentheses, using the standard amino acid three-letter code. VisGA, VisGB, VisGC represent NRPS enzymes for viscosin-group CLPs that have been characterized based on genome mining only. The phylogenetic tree was drawn using the Geneious 11.1.5 software. Clusters are shown according to the type of C domains in color; *N*-acylating C_s_ starter domains (orange), conventional ^L^C_L_ domains (pink), dual condensation/epimerization domains (blue). The phylogenetic tree was drawn using the Geneious 11.1.5 software.

*In silico* analysis of the C-domains revealed the presence of a lipo-initiation (C starter) domain in module 1 of VsmA and PdmA, indicating that a fatty acid is attached to Leu1. Furthermore, it revealed that module 8 has a conventional ^L^C_L_-domain while the rest of the C-domains are of the C/E-type (Figure A3). According to the Balibar rule [45], this is consistent with the presence of l-Leu7 but suggests that the C-domain of module 2 in Viscosin-group NRPSs lacks general epimerization activity on l-Leu1. The latter also applies to the 6th module of viscosinamide, viscosin, and massetolide (all with l-Leu5) but not for pseudodesmin and WLIP (both with d-Leu5). The termination module of *pdmC* and *vsmC* consists of tandem TE-domains. Phylogenetic analysis showed that the first and second TE-domain consistently cluster with the Type I and Type II TE-domains of *Pseudomonas* CLPs, respectively (Figure S3).

### 3.3. Phylogenetic Analyses of CLP-Producing Strains within the Viscosin Group

**Figure 5:**
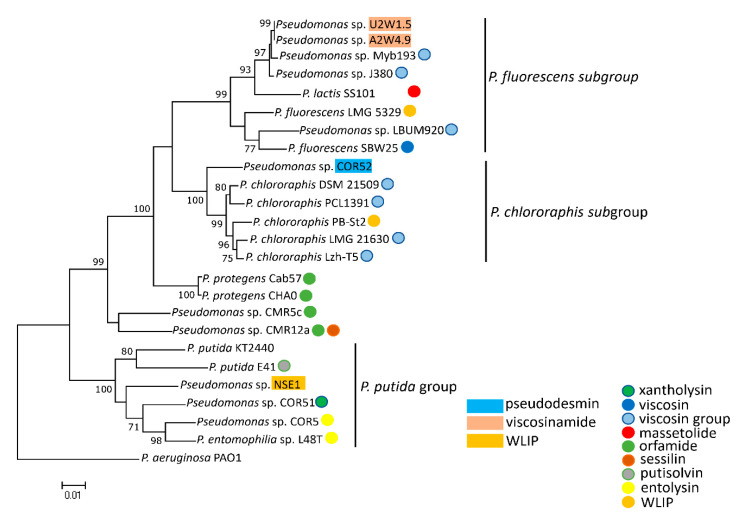
MLSA-derived phylogenetic analyses of *Pseudomonas* isolates carrying Viscosin-group BGCs. Partial sequences of 16S rRNA, *gyrB*, *rpoB* and *rpoD* genes were used to make separate trees and concatenated afterwards, to generate a consensus tree. Other biocontrol CLP-producing isolates and type strains were included in this analysis. *P. aeruginosa* was used as an outgroup. A maximum likelihood tree was made and bootstraps are only indicated for branches with bootstrap support of higher than 70%. CLPs from strains denoted in rectangular, colored boxes were described in this study, while those marked with color-filled dots were characterized in previous studies, except for light blue dots representing putative Viscosin group producers for which the CLP has not been characterized.

In line with the A-domain phylogeny, MLSA analysis situated the pseudodesmin and viscosinamide producers within the *P. chlororaphis* and *P. fluorescens* subgroups, respectively (Figure 5). This taxonomic-biosynthetic congruence is also seen for the putative viscosinamide producers Myb193 and J380, close relatives of strains A2W4.9 and U2W1.5. Although strain COR52 belongs to the *P. chlororaphis* subgroup, it is located on a separate branch, which is reflected in the production of pseudodesmin (COR52) versus WLIP (Pb-St2) that differ only by one residue (l-Gln2 and l-Glu2).

### 3.4. Swarming Motility and White Line-in-Agar Assay

**Figure 6:**
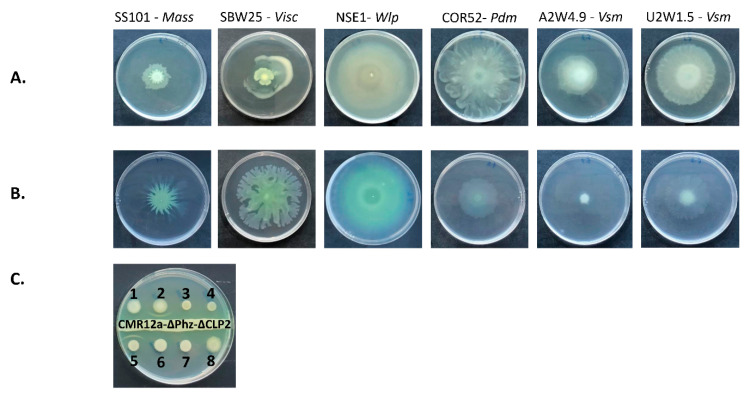
Swarming and white line-in-agar phenotype for *Pseudomonas* strains producing viscosin-group CLPs. Swarming phenotype of strains grown on (**A**) 0.6% LB medium and (**B**) 0.6% Standard Succinate Medium (SSM). (**C**) White line-in-agar test conducted for Viscosin-group CLP producers in interaction with sessilin producer, *Pseudomonas* sp. CMR12a-ΔPhz-ΔCLP2. Strains are labelled 1–8: (1) *Pseudomonas* sp. COR52 (pseudodesmin), (2) *Pseudomonas* sp. NSE1 (WLIP), (3) *P. lactis* SS101 (massetolide), (4) *P. fluorescens* SBW25 (viscosin), (5) *P. protegens* Pf-5 (orfamide), (6) *Pseudomonas* sp. A2W4.9 (viscosinamide), (7) *Pseudomonas* sp. U2W1.5 (viscosinamide), (8) *P. fluorescens* DR54 (viscosinamide).

Motility tests were carried out using 0.6% LB and 0.6% SSM agar. Each CLP-producing strain within the Viscosin group gave a unique swarming phenotype (Figure 6A,B). On LB media, the WLIP producer (NSE1), pseudodesmin producer (COR52) and the viscosinamide producer from Umudike (U2W1.5) swarmed fairly well. The viscosinamide producer from Ado-Ekiti (A2W4.9) swarmed moderately and comparably with the viscosin producer (SBW25), while the massetolide-producing strain swarmed least. On SSM media, the WLIP, massetolide and viscosin producers displayed good swarming phenotypes. In contrast, pseudodesmin and viscosinamide producers showed less swarming motility on this medium. All these CLP-producing strains belonging to the Viscosin group were also tested for white line-in-agar interaction with the sessilin producer, CMR12a-ΔPhz-ΔCLP2. Only the WLIP producer (NSE1) and the positive control strain *P. protegens* Pf-5, which produces orfamide, gave a white line interaction (Figure 6C).

### 3.5. Inhibition of P. myriotylum by Pseudodesmin, Viscosinamide and WLIP

Given that these two CLPs have not been widely reported until now, it is worthwhile to investigate their probable biological impact on root pathogens. Therefore, we compared the biological activity profile of these CLPs on a pathogen of their natural host (*P. myriotylum*) and a pathogen that was isolated from a bean in a tropical environment in Cuba [43], *R. solani* AG2-2.

Bioactivity tests against *R. solani* AG2-2 and *P. myriotylum* CMR1 were carried out with various concentrations of purified pseudodesmin and viscosinamide, while purified WLIP was used for comparison. Arrows on microscopic figures indicate the spot where the CLP drop was placed.

**Figure 7:**
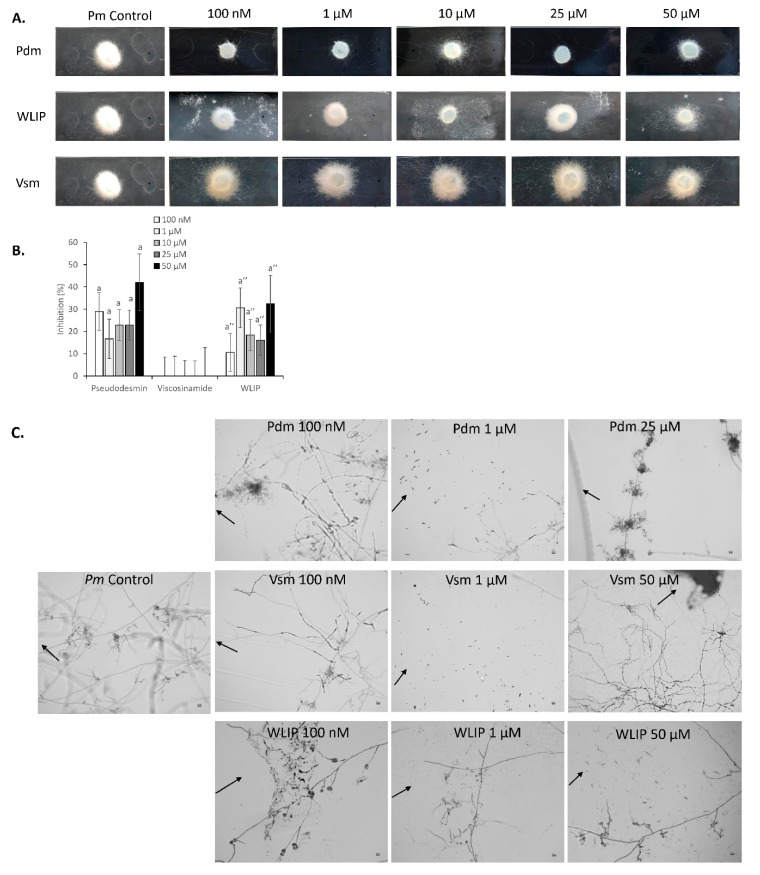
Effect of pseudodesmin, viscosinamide and WLIP on the mycelial growth and hyphal morphology of *P. myriotylum* CMR1. (**A**) Effect of various CLP treatments and concentrations on the growth of *P. myriotylum* CMR1 (Pm). For each CLP, concentrations of 100 nM, 1 µM, 10 µM, 25 µM and 50 µM were tested. Representative images are shown. (**B**) Percentage inhibition of *P. myriotylum* CMR1 in response to various CLP treatments and concentrations. Values indicate percentage growth inhibition relative to the control (*n* = 5). The data were analyzed using Tukey’s post-hoc tests via SPSS statistics 25. Bars with different letters indicate significant differences (*p* < 0.05) between treatments; vertical lines indicate the standard error (SE). The letters a and a’ were used to denote statistics within treatments for pseudodesmin and WLIP respectively. (**C**) Microscopic assays showing the effect of various CLP treatments and concentrations on the mycelia of *P. myriotylum* CMR1. Arrows indicate the position of the CLP droplets.

Exposure of *P. myriotylum* to purified pseudodesmin significantly inhibited pathogen growth at all concentrations tested (Figure 7A,B). In contrast, the application of all the concentrations of viscosinamide enhanced the growth of *P. myriotylum* mycelium without any visible inhibition zone. WLIP treatments gave visual inhibition zones that could not be easily measured considering the fact that the pathogen appeared to grow around the spot containing the CLP. In the presence of WLIP, *P. myriotylum* appeared to grow denser than with the other treatments, but evaded the spot where the compound was introduced.

### 3.6. P. myriotylum-CLP Perception and Interaction

Treatment of *P. myriotylum* with 100 nM, 10 µM and 25 µM of pseudodesmin led to increased/hyper branching, hyphal distortion and CLP evasion (Figure 7C, Table A2). In treatments with 1 and 50 µM pseudodesmin, there was hyphal disintegration/distortion and reduced hyphal branching. In treatments with viscosinamide, however, all concentrations except 50 µM showed hyphal distortion and lysis. In contrast, 50 µM of viscosinamide resulted in the enhanced elongation of primary mycelium and the formation of convergent structures by mycelia. Furthermore, treatments with WLIP showed hyphal disintegration, timely evasion and an absence of branching (Figure 7C).

### 3.7. Inhibition of R. solani by Pseudodesmin, Viscosinamide and WLIP

**Figure 8:**
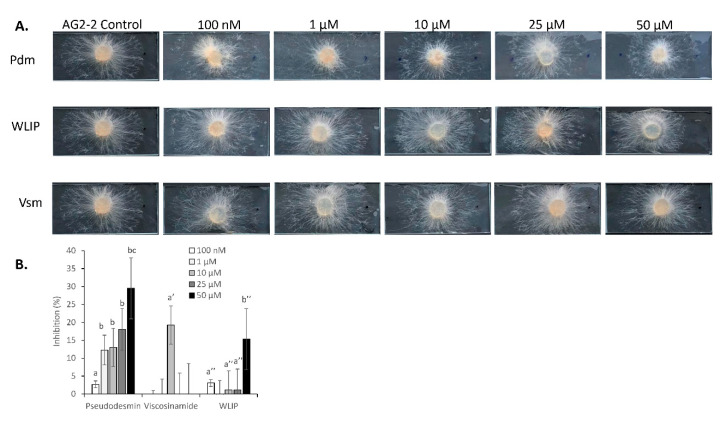

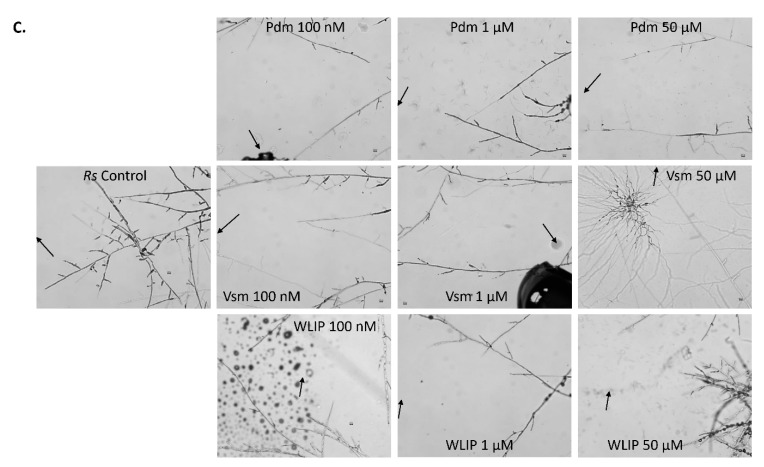
In vitro bioactivity assays showing the effect of pseudodesmin, viscosinamide and WLIP on the mycelial growth and hyphal morphology of *R. solani* AG 2-2. (**A**) Effect of various CLP treatments and concentrations on the growth of *R. solani* AG 2-2. For each CLP, concentrations of 100 nm, 1 µM, 10 µM, 25 µM and 50 µM were tested. Representative images are shown. (**B**) Percentage inhibition of *R. solani* AG 2-2 in response to various CLP treatments and concentrations. Values indicate percentage growth inhibition relative to the control (*n* = 5). The data were analyzed using Tukey’s post-hoc tests via SPSS statistics 25. Bars with different letters indicate significant differences (*p* < 0.05) between treatments; vertical lines indicate the standard error (SE). The letters a/b, a’ and a’’/b’’ were used to denote statistics within treatments for pseudodesmin, viscosinamide and WLIP respectively. (**C**) Microscopic assays showing the effect of various CLP treatments and concentrations on the mycelia of *R. solani* AG2-2. Arrows indicate the position of the CLP droplets.

In tests with *R. solani* AG 2-2, 100 nM of pseudodesmin gave a visual inhibition zone which became enhanced with higher concentrations of 1, 10, 25 and 50 µM (Figure 8A,B, Table A2). In contrast, no visual inhibition zone was observed in treatments with viscosinamide except at 10 µM. WLIP mainly gave a slight inhibition zone at the lowest (100 nM) concentration and a strong inhibition at the highest concentration (50 µM) tested, while the effects of the intermediate concentrations were unclear.

### 3.8. R. solani-CLP Perception and Interaction

In tests involving *R. solani*, concentrations of pseudodesmin (0.1, 1, 10, 25 and 50 µM) showed CLP evasion by the pathogen, hyphae distortion and the disintegration of hyphae with debris observed on the slide (Figure 8C, Table A2). All concentrations of viscosinamide tested except 50 µM showed CLP evasion, hyphal distortion and the rupturing of contents. At 50 µM, mycelia distortion and disintegration was observed in combination with a coordinated formation of melanized structures by multiple primary hyphae as they approached the CLP droplet. In treatments with WLIP, *R. solani* hyphae showed increased branching as they grew in the direction of the compound. Upon contact with the compound, the disintegration of hyphae was observed (Figure 8C).

## 4. Discussion

In this study, we conducted the biological and genetic characterization of two CLPs from cocoyam rhizosphere isolates, identified as pseudodesmin and viscosinamide produced by *Pseudomonas* spp. COR52 and A2W4.9, respectively.

### 4.1. CLP Identification and in silico Analysis of Pseudodesmin and Viscosinamide Genes

Using NMR, we identified the respective main CLP metabolites as viscosinamide A and pseudodesmin A, which differ only in the stereochemistry of the fifth amino acid (l-Leu5 for viscosinamide A and d-Leu5 for pseudodesmin A). In this study, there was a perfect overlay of chemical shift values between those of the described viscosinamide producer DR54 and those obtained from A2W4.9. Similar results were obtained when the chemical shifts of synthetic pseudodesmin were compared with those of the COR52 compound.

Subsequently, we identified the BGCs of pseudodesmin and viscosinamide in strains *Pseudomonas* spp. COR52 and A2W4.9, respectively. Apart from the uncertain assignment of the second amino acid (Gln *versus* Glu), the predicted peptide sequences match well with the experimentally determined structures [17,18]. Prior to this work, biosynthetic gene clusters of Viscosin-group CLPs that have so far been described have three NRPS genes that occur in separate operons; a first operon comprising gene A and a second operon that contains genes B and C [24,46]. Our study reports a similar genetic organization in the viscosinamide gene cluster. However, the genetic backbone of the single pseudodesmin gene cluster deviates from this genomic arrangement and is similar to those obtained in CLPs, such as arthrofactin, anikasin, and lokisin [25] and many others. Based on BGC characteristics and MLSA, the characterized CLP producer most related to pseudodesmin producer COR52 is WLIP producer *P. chlororaphis* Pb-St2 [28]. Interestingly, the similar BGC organization of multiple other *P. chlororaphis* strains hints to the production of a viscosin-type CLP. Whether these strains actually produce pseudodesmin, WLIP, or yet another CLP of the Viscosin group remains to be clarified chemically. Furthermore, our study again raises the question about why some CLP NRPSs are physically linked and others have the initiatory NRPS gene in a remote operon. A separate location of these operons may suggest additional functions by the initiatory NRPS gene besides CLP production within the producing strain.

### 4.2. Taxonomic Congruence and Functional Analyses of Pseudodesmin- and Viscosinamide-Producing Strains

Phylogenetic MLSA analyses showed that the cocoyam pseudodesmin and viscosinamide producers are associated with the *P. chlororaphis* and *P. fluorescens* subgroups, respectively, while WLIP producer NSE1 belongs to the *P. putida* group. However, WLIP producers have also been reported in the *P. fluorescens* subgroup [47] and the *P. chlororaphis* subgroup. *P. fluorescens* LMG 5329, was isolated from decaying sporocarps of oyster mushrooms [48] and belongs to the *P. fluorescens* subgroup. *P. chlororaphis* PB-St2 was obtained from the stem of sugarcane. Hence, the *P. fluorescens* group (delineated according to [49]) comprises multiple producers of the Viscosin group of CLPs, namely pseudodesmin (*P. chlororaphis* subgroup), massetolide, viscosin, viscosinamide (*P. fluorescens* subgroup) and WLIP (*P. fluorescens* and *P. chlororaphis* subgroup). This is perhaps unsurprising since all Viscosin-group CLPs are closely related and mainly differ from each other by the presence of a Glu or Gln at peptide position 2 and some merely by the d/l-configuration of one amino acid residue. With the exception of the WLIP, the congruence between the phylogenetic relationship of strains and the CLPs they produce indicates evolutionary divergence within these separate hosts. Besides antimicrobial functions, it has been suggested that CLP diversity may be driven by microbial competition [8]. Worthy of note is the capacity of the WLIP producers, NSE1 and RW10S2, to interact with tolaasin and the tolaasin-like CLP sessilin to give a white line while other Viscosin group members do not. In our previous study, the chemical analysis of the white line formed by the co-inoculation of the sessilin producer of CMR12a and the orfamide producer (Pf-5) revealed the presence of both CLPs in the white line compound [41]. Thus, this suggests that the secretion of WLIP into the environment and its activity can be potentially interfered with by competing organisms. Thus, we opine that these competing interactions drive CLP evolution and diversity. Hence, several strains tend to produce closely related CLPs. In this context, it should be noted that the WLIP producer *P. chlororaphis* Pb-St2 did not form a white line with a tolaasin producer [28]. The reason for this is unknown.

### 4.3. Swarming Motility Rates in Different Media

With the exception of the WLIP producer (NSE1), swarming motility patterns and rates differed depending on the media. Compared with pseudodesmin (COR52) and viscosinamide producers (A2W4.9 and U2W1.5), the viscosin producer SBW25 and to some extent, the massetolide producer SS101, swarmed remarkably well on SSM medium. In previous studies, the production of massetolide and viscosin has been shown to be important for the swarming motility of SS101 and SBW25, respectively [22,23]. While we cannot exclude the role of other factors in the observed swarming motility of these strains, the role of CLPs in this phenomenon remains to be investigated for other strains used during this study. Our results suggest that, under different nutrient conditions (SSM) other than LB, viscosinamide- and pseudodesmin-producing strains swarm less. It remains to be investigated whether this is due to reduced CLP production or a down-signaling of other factors that may likely be involved in motility. Knowledge of what drives this phenomenon within this CLP group will advance our understanding of these CLPs and their functionality in their specific niche.

### 4.4. CLP Rarity vs. Functional Relevance

This study focused on two CLPs that are considered to be comparatively rare since their occurrence has previously only been reported once and orthologous BGCs are found in only a limited number of *Pseudomonas* genomes. With respect to origin, the only viscosinamide producer previously reported (strain DR54) was isolated from the rhizosphere of 7-day old sugar beet [17]. Our study reports two other viscosinamide producers that originate from the 2-week old tissue culture-derived cocoyam plantlets. Thus, viscosinamide producers may be associated with young plants and their recruitment may be dependent on root exudate composition. On the other hand, our viscosinamide BGCs cluster closely with those of two putative viscosinamide producers (MYb193 and J380). These two strains were obtained from compost and cunner fish sources, respectively, and it remains to be proven experimentally that they actually produce viscosinamide. As for pseudodesmin, the first reported producer, identified as *P. tolaasii* but no longer being available (personal communication, José C. Martins), was isolated from the mucus skin layer of the black belly salamander [18]. The closest related strains to our pseudodesmin producer (COR52) all belong to the *P. chlororaphis* subgroup and are situated in different subspecies and in diverse ecological niches, including various plants and fish [50]. At least one of these strains, *P. chlororaphis* Pb-St2, which originates from a surface sterilized stem of sugarcane produces WLIP [28]. Chemical analysis will be needed to verify whether the other *P. chlororaphis* strains produce WLIP, pseudodesmin, or yet another CLP of the Viscosin group. The presence of pseudodesmin and viscosinamide producers on tissue culture-derived young cocoyam roots is in contrast with the presence of pseudomonads producing different CLP types on field-grown cocoyam roots in Cameroon [8,11] and Nigeria [8]. It raises the question about the probable effect of plant age and root exudation composition on CLP diversity, which is part of our current research.

### 4.5. Biological Activity of Pseudodesmin, Viscosinamide and WLIP against P. myriotylum—Mechanism of Action?

Varying concentrations of pseudodesmin and viscosinamide appeared to stimulate the hyphal branching and disintegration of *P. myriotylum,* whereas nanomolar amounts of WLIP were sufficient to block hyphal branching and led to mycelia disintegration. It was also striking to observe the hyperbranching phenotype in treatments with nano- and micromolar concentrations of pseudodesmin. Similar phenotypes are caused by morphogenic plant defensins, compounds that play important roles in plant defense. At micromolar concentrations, they are able to induce hyphal elongation and hyperbranching in invading fungi [51,52]. The mechanism of action of the morphogenic plant defensin *Medicago sativa* defensin 1 (MsDef1) has been attributed to its capacity to disrupt Ca^2+^ signaling and Ca^2+^ gradients in the hyphal tips leading to hyperbranching [53]. Whether the antifungal activity of pseudodesmin (and perhaps other CLPs) is calcium channel mediated is worth exploring further.

Viscosinamide and pseudodesmin CLPs are neutral, have similar amino acid (AA) composition and only differ from each other by the stereochemistry on the 5th AA [30]. The difference in activity observed here may suggest an effect of this stereochemical difference. A recent study tested the antibacterial activity of 20 novel synthesized pseudodesmin A analogs and reported a mode of action determined by the macrocycle closed by an ester bond and the critical length of the β-OH fatty acid chain capping the N-terminus [31]. Bioactivity tests of six Gram-positive bacterial strains showed equal activity of pseudodesmin and viscosinamide, suggesting that the stereochemical shift of a d-Leu to a l-Leu had no consequence [31]. It was striking to observe that for all treatments and concentrations, *P. myriotylum* evaded the CLP except at 50 µM viscosinamide, where conglomerate structures were formed probably in response to CLP-induced stress.

### 4.6. Perception of CLPs by P. myriotylum—Implications for CRRD Suppression

The observations of WLIP evasion by *P. myriotylum* subsequent to mycelia damage suggests that there is no early perception of this CLP by the pathogen. This appears to be the case because *P. myriotylum* grew evenly on sides with or without the CLP with a visible evasion response only after contact with the compound. Fungi can sense environmental signals and respond accordingly either by changing their development, direction of growth, and metabolism [54]. The capacity for sensory perception enables fungi to know when and how to infect a plant host. What is observed here may be similar to the capacity of fungi to detect bacterial presence through Microbe-Associated Molecular Patterns (MAMPs) [55]. In an environment replete with pathogenic and beneficial organisms, survival is dependent on efficient pathogen sensing and the capacity to rapidly mount defense responses. Thus, this is similar to the protective mechanism of innate immunity that is found in multicellular organisms [55]. The differential CLP behaviors observed in this study lend a twist to our understanding regarding their roles in CRRD suppression in the cocoyam rhizosphere that was previously reported [56]. Besides a sexual cycle, *P. myriotylum* has an asexual reproductive cycle that consists of mycelium, which produces sporangia [42,57]. These sporangia are terminal and intercalary, filamentous, consisting of undifferentiated and inflated lobulated or digitate elements of variable length. Our study shows that WLIP completely blocks the formation of branching and eventually disintegrates the entire mycelia. This information is intriguing and suggests that an abundance of WLIP producers in the cocoyam rhizosphere may correlate to CRRD suppressiveness. This may not be clear-cut since WLIP producers were identified from the cocoyam rhizosphere in five different fields in Cameroon and Nigeria, in both suppressive and conducive soils [8]. It is plausible that a consortium of strains producing CLPs that cannot be perceived by the target pathogen, as with the WLIP, may produce an arsenal of metabolites against *P. myriotylum*. The behavior of pseudodesmin and viscosinamide in interaction with *P. myriotylum* gives insights into the adaptation and responses of *P. myriotylum* in interaction with different CLPs that ultimately result in pathogen lysis or the evasion of the CLP. The probable concerted action of cocoyam-associated CLPs obtained from CRRD-suppressive soils vis-à-vis *P. myriotylum* should be investigated further.

### 4.7. Differential Responses of R. solani to Pseudodesmin, Viscosinamide and WLIP

Furthermore, experiments involving *R. solani*, pseudodesmin and viscosinamide also gave different responses. Although applications of all pseudodesmin concentrations resulted in the evasion or disintegration of *R. solani* hyhae, this effect was replicated in viscosinamide treatments except at 50 µM, where convergent melanized structures were formed probably in response to CLP-induced stress. Thus for both pathogens tested during this study, higher concentrations of viscosinamide appeared to evoke a stress response in the pathogen, thereby resulting in the formation of survival (and/or melanized) structures. Viscosinamide was shown to induce physiological changes in *P. ultimum*, *P. oligandrum* and *R. solani* under in vitro and soil conditions [32,58]. The intracellular change in hyphae mediated by viscosinamide is likely due to its capacity to form ion channels in the cell membrane [32]. In this previous study, although *R. solani* AG4 was used in contrast with our study in which AG2-2 was used, it appears that viscosinamide shows antimicrobial activity against this pathogen irrespective of anastomosis group (AG) affiliation. Moreover, different concentrations of viscosinamide were not tested in the previous study; hence, a direct comparison of results is not feasible. It is also important to highlight that evaluating the response of plant pathogens to high concentrations of pseudodesmin and viscosinamide may not be physiologically relevant since these compounds are known to predominantly accumulate in the bacterial cell wall [17] (Geudens, N., personal communication). In general, regarding the assessment of biological activity, we observed that the measurement of a visual inhibition zone is not an optimal method for the assessment of the biological activity of these compounds since the absence of a visual inhibition did not correlate with inhibitory effects observed via microscopy.

Similar with our observation in *P. myriotylum* experiments, *R. solani* appeared not to recognize WLIP but grew towards it until hyphal disintegration occurred. In this study, it appears that the perception and bioactivity of viscosinamide and pseudodesmin by *P. myriotylum* and *R. solani* may be dictated by their lack of charge due to the presence of a Gln2. In contrast, WLIP has a negative charge due to the presence of a Glu2. Our studies indicate that, at specific concentrations, this CLP was perceived and evaded by *R. solani* but not by *P. myriotylum*. It remains to be seen if viscosin, a CLP that only differs from WLIP by the stereochemistry on the 5th amino acid, will show similar responses and bioactivity to that observed with WLIP. Thus, our studies highlight discordant perception patterns of these members of the Viscosin group in relation to these two plant pathogens. This knowledge will be explored further in the understanding of microbe–plant–CLP interactions.

## 5. Conclusions

In summary, our study scrutinizes the biosynthetic machinery of the viscosinamide and pseudodesmin CLPs and how they differ bioinformatically in comparison to other members of the Viscosin group of CLPs. Pseudodesmin and viscosinamide producers were obtained from young tissue culture-derived cocoyam plantlets and it is worth investigating whether their presence there is driven by root exudate composition. Among all the CLP-producing strains tested during this study, WLIP appears to possess the highest surface-active property in view of its strong capacity to promote swarming motility. Besides, this CLP is able to successfully antagonize *R. solani* and *P. myriotylum*, by evading recognition until hyphal disintegration occurs. On the other hand, both pseudodesmin and viscosinamide cause extensive branching in *P. myriotylum* and subsequent lysis or CLP evasion, while *R. solani* evaded these CLPs. Thus, CLPs may function as MAMPs that can be recognized by pathogens, but this obviously needs further investigation. Our future studies aim to elaborate further on the natural roles of structurally diverse CLPs in plant–pathogen interactions.

## Supplementary Materials

The following are available online at https://www.mdpi.com/2076-2607/8/7/1079/s1, Figure S1: (A) 1D ^1^H NMR spectrum of major isolated CLP (N6, pseudodesmin A) (500 MHz, CD_3_CN); (B) The alpha region of a ^1^H-^13^C gHSQC spectrum of the isolated CLP; Figure S2: (A) 1D ^1^H NMR spectrum of major isolated CLP (viscosinamide A) (500 MHz, CD_3_CN); (B) The alpha region of a ^1^H-^13^C gHSQC spectrum of the isolated CLP; Figure S3: Phylogenetic analyses of the Te domains extracted from NRPSs belonging to the Viscosin Group; Table S1: ^1^H and ^13^C chemical shift values of the isolated compound (N6, pseudodesmin A) (500 MHz, CD_3_CN, 298K); Table S2: ^1^H and ^13^C chemical shift values of the isolated compound (viscosinamide A) (500 MHz, CD_3_CN, 298K).

## Appendix A

**Table A1:** List of strains and genomes used during this study and their relevant characteristics.

Species	Strain Name	CLP Produced *	Origin/Source	Host	Accession	Country	Reference
*P. chlororaphis* subsp. *piscium*	PCL1391	viscosin G ^1^	Tomato rhizosphere	*Solanum lycopersicum*	CP027736.1	Spain	[50]
*P. chlororaphis* subsp. *piscium*	DSM 21509	viscosin G ^1^	European perch intestine	*Perca fluviatilis*	CP027707.1	Switzerland	[50]
*P. chlororaphis* subsp. *aureofaciens*	P2	viscosin G ^1^	Potato roots	*Solanum tuberosum*	CP027719.1	Algeria	[50]
*P. chlororaphis*	Pb-St2	WLIP	Sugarcane stem	*Saccharum* sp.	CP027716.1	Pakistan	[50]
*P. chlororaphis* subsp. *aurantiaca*	PCM 2210	viscosin G ^1^	Sugar beet roots	*Beta vulgaris*	CP027717.1	Poland	[50]
*P. chlororaphis* subsp. *piscium*	ZJU60	viscosin G ^1^	-	Wheat	CP027656.1	China	Chen and Ma, unpublished
*P. chlororaphis*	Lzh-T5	viscosin G ^1^	Soil	-	CP025309.1	China	Li et al., unpublished
*Pseudomonas* sp.	MYb193	viscosin G ^2^	Compost	-	CP023269.1	Germany	[59]
*Pseudomonas* sp.	J380	viscosin G ^2^	-	Cunner fish	CP043060.1	Canada	Santander et al., unpublished
*P. synxantha*	2-79	viscosin G ^3^	Wheat rhizosphere	*Triticum* sp.	CP027755.1	USA	[50]
*Pseudomonas* sp.	LBUM920	viscosin G ^3^	White spruce rhizosphere	*Picea glauca*	CP027762.1	Canada	[50]
*P. antarctica*	PAMC 27494	viscosin G ^3^	Freshwater	-	CP015600.1	Antarctica	Lee, unpublished
*P. lactis*	SS101	massetolide	Wheat rhizosphere	*Triticum* sp.	CM001513.1	Netherlands	[23]
*P. synxantha*	30B	viscosin G ^3^	Wheat rhizosphere	*Triticum* sp.	CP027754.1	Iran	[50]
*P. putida*	RW10S2	WLIP	Rice rhizosphere	*Oryza sativa*	JN982332.1	Sri Lanka	[24]
					*(wlipA)*		
					JN982333.1 *(wlipBC)*		
*Pseudomonas sp.*	NSE1	WLIP	white cocoyam rhizosphere	*X. sagittifolium*	MK534106.1	Nigeria	[25]
					*(wlipA)*		
					MK650230.2		
					*(wlipBC)*		
*P. fluorescens*	SBW25	viscosin	Wheat rhizosphere	*Triticum* sp.	NC_012660.1	Netherlands	[60]
*Pseudomonas* sp.	COR52	pseudodesmin	tissue cultured red cocoyam	*X.. sagittifolium*	MT577358	Cameroon	This study
*Pseudomonas* sp.	U2W1.5	viscosinamide	tissue cultured white cocoyam	*X. sagittifolium*	MT771986	Nigeria	This study
					*(vsmA)*		
					MT749673		
					*(vsmBC)*		
*Pseudomonas* sp.	A2W4.9	viscosinamide	tissue cultured white cocoyam	*X. sagittifolium*	MT749674	Nigeria	This study
					*(vsmA)*		
					MT771985		
					*(vsmBC)*		

G = Group, *X. sagittifolium* = *Xanthosoma sagittifolium.* * Viscosin G denotes CLPs that have been predicted by genome mining only without chemical structure analysis. ^1^ putative pseudodesmin or WLIP producers; no bipartite BGC and similar to *P. chlororaphis* COR52 and *P. chlororaphis* Pb-St2. ^2^ putative viscosinamide producers; bipartite BGCs clusters closely with *Pseudomonas* sp. A2W4.9 and U2W1.5. ^3^ putative viscosin producers; bipartite BGC clusters closely with *P. fluorescens* SBW25.

**Table A2:** Comparison between the effects of pseudodesmin, viscosinamide and WLIP on *P. myriotylum* and *R. solani* AG2-2.

CLP	Concentration	Growth Inhibition	Hyphal Blockage	Hypal Distortion	Hyphal Branching	Hyper Branching	Hyphal Disintegration	Hyphal Lysis	CLP Evasion	Convergent Structures *
pseudodesmin	100 nM	+/+	−/+	++/+	++/−	++/−	−/−	−/+	−/+	−/−
	1 µM	+/+	−/+	++/+	++/−	−/−	−/−	++/+	−/+	−/−
	10 µM	+/+	−/+	++/+	++/−	−/−	−/−	−/+	+/+	−/−
	25 µM	+/+	−/+	++/+	+/−	++/−	−/−	−/+	+/+	−/−
	50 µM	++/++	−/+	++/+	−/−	−/−	−/−	−/+	−/+	−/−
viscosinamide	100 nM	−/−	−/+	+/+	++/−	−/−	−/−	−/−	−/+	−/−
	1 µM	−/−	−/+	+/+	++/−	−/−	−/−	+/−	−/+	−/−
	10 µM	−/+	−/+	+/+	+/−	−/−	−/−	−/−	+/+	−/−
	25 µM	−/−	−/+	+/+	++/−	−/−	−/−	+/−	−/+	−/−
	50 µM	−/−	−/+	+/+	++/−	−/−	−/−	−/−	+/+	++/++
WLIP	100 nM	+/+	+/+	+/+	−/−	−/−	+/+	−/−	+/−	−/−
	1 µM	+/−	+/+	+/+	−/−	−/−	+/+	−/−	+/−	−/−
	10 µM	+/+	+/+	+/+	−/−	−/−	++/+	−/−	+/−	−/−
	25 µM	+/+	+/+	+/+	−/−	−/−	++/++	−/−	+/−	−/−
	50 µM	++/++	+/+	+/+	−/−	−/−	++/++	−/−	+/−	−/−

For each CLP, each parameter and concentration, the first score is for tests with *P. myriotylum* CMR1 while the second is for tests with *R. solani* AG2-2. * melanization of convergent mycelial structures were observed for tests with *R. solani* AG2-2.

## References

[1] Raaijmakers J.M., de Bruijn I., de Kock M.J.D. (2006). Cyclic lipopeptide production by plant-associated *Pseudomonas* spp.: Diversity, activity, biosynthesis, and regulation. Mol. Plant. Microbe Interact..

[2] Geudens N., Martins J.C. (2018). Cyclic lipodepsipeptides from *Pseudomonas* spp.–biological swiss-army knives. Front. Microbiol..

[3] Raaijmakers J.M., Mazzola M. (2012). Diversity and natural functions of antibiotics produced by beneficial and plant pathogenic bacteria. Ann. Rev. Phytopathol..

[4] Jang J.Y., Yang S.Y., Kim Y.C., Lee C.W., Park M.S., Kim J.C., Kim I.S. (2013). Identification of orfamide A as an insecticidal metabolite produced by *Pseudomonas protegens* F6. J. Agric. Food Chem.

[5] Cautain B., de Pedro N., Schulz C., Pascual J., Sousa T.D., Martin J., Perez-Victoria I., Asensio F., Gonzalez I., Bills G.F. (2015). Identification of the lipodepsipeptide MDN-0066, a novel inhibitor of vhl/hif pathway produced by a new *Pseudomonas* species. PLoS ONE.

[6] Olorunleke F.E., Kieu N.P., Höfte M., Murillo J., Vinatzer B.A., Jackson R.W. (2015). Recent advances in *Pseudomonas* biocontrol. Bacteria-Plant Interactions: Advance Research and Future Trends.

[7] Flury P., Vesga P., Pechy-Tarr M., Aellen N., Dennert F., Hofer N., Kupferschmied K.P., Kupferschmied P., Metla Z., Ma Z.W. (2017). Antimicrobial and insecticidal: Cyclic lipopeptides and hydrogen cyanide produced by plant-beneficial *Pseudomonas* strains CHA0, CMR12a, and PCL1391 contribute to insect killing. Front. Microbiol..

[8] Oni F.E., Geudens N., Onyeka J.T., Olorunleke O.F., Salami A.E., Omoboye O.O., Arias A.A., Adiobo A., De Neve S., Ongena M. (2020). Cyclic lipopeptide-producing *Pseudomonas koreensis* group strains dominate the cocoyam rhizosphere of a Pythium root rot suppressive soil contrasting with *P. putida* prominence in conducive soils. Environ. Microbiol..

[9] Gotze S., Stallforth P. (2020). Structure, properties, and biological functions of nonribosomal lipopeptides from pseudomonads. Nat. Prod. Rep..

[10] Jahanshah G., Yang Q., Gerhardt H., Pataj Z., Lammerhofer M., Pianet I., Josten M., Sahl H.G., Silby M.W., Loper J.E. (2019). Discovery of the cyclic lipopeptide gacamide A by genome mining and repair of the defective gacA regulator in *Pseudomonas fluorescens* Pf0-1. J. Nat. Prod..

[11] Oni F.E., Geudens N., Omoboye O.O., Bertier L., Hua H.G.K., Adiobo A. (2019). Fluorescent *Pseudomonas* and cyclic lipopeptide diversity in the rhizosphere of cocoyam (*Xanthosoma sagittifolium*) (vol 21, pg 1019, 2019). Environ. Microbiol..

[12] Gross H., Loper J.E. (2009). Genomics of secondary metabolite production by *Pseudomonas* spp. Nat. Prod. Rep..

[13] Ziemert N., Alanjary M., Weber T. (2016). The evolution of genome mining in microbes-a review. Nat. Prod. Rep..

[14] Roongsawang N., Washio K., Morikawa M. (2011). Diversity of nonribosomal peptide synthetases involved in the biosynthesis of lipopeptide biosurfactants. Int. J. Mol. Sci..

[15] Ziemert N., Jensen P.R. (2012). Phylogenetic approaches to natural product structure prediction. Methods Enzymol..

[16] Laycock M.V., Hildebrand P.D., Thibault P., Walter J.A., Wright J.L.C. (1991). Viscosin, a potent peptidolipid biosurfactant and phytopathogenic mediator produced by a pectolytic strain of pseudomonas-fluorescens. J. Agric. Food Chem.

[17] Nielsen T.H., Christophersen C., Anthoni U., Sorensen J. (1999). Viscosinamide, a new cyclic depsipeptide with surfactant and antifungal properties produced by *Pseudomonas fluorescens* DR54. J. Appl. Microbiol..

[18] Sinnaeve D., Michaux C., Van Hemel J., Vandenkerckhove J., Peys E., Borremans F.A.M., Sas B., Wouters J., Martins J.C. (2009). Structure and x-ray conformation of pseudodesmins A and B, two new cyclic lipodepsipeptides from *Pseudomonas* bacteria. Tetrahedron.

[19] Pedras M.S.C., Ismail N., Quail J.W., Boyetchko S.M. (2003). Structure, chemistry, and biological activity of pseudophomins A and B, new cyclic lipodepsipeptides isolated from the biocontrol bacterium *Pseudomonas fluorescens*. Phytochemistry.

[20] Gerard J., Lloyd R., Barsby T., Haden P., Kelly M.T., Andersen R.J. (1997). Massetolides A-H, antimycobacterial cyclic depsipeptides produced by two pseudomonads isolated from marine habitats. J. Nat. Prod..

[21] Mortishiresmith R.J., Nutkins J.C., Packman L.C., Brodey C.L., Rainey P.B., Johnstone K., Williams D.H. (1991). Determination of the structure of an extracellular peptide produced by the mushroom saprotroph *Pseudomonas-reactans*. Tetrahedron.

[22] De Bruijn I., de Kock M.J.D., de Waard P., van Beek T.A., Raaijmakers J.M. (2008). Massetolide A biosynthesis in *Pseudomonas fluorescens*. J. Bacteriol..

[23] De Bruijn I., de Kock M.J.D., Yang M., de Waard P., van Beek T.A., Raaijmakers J.M. (2007). Genome-based discovery, structure prediction and functional analysis of cyclic lipopeptide antibiotics in *Pseudomonas* species. Mol. Microbiol..

[24] Rokni-Zadeh H., Li W., Sanchez-Rodriguez A., Sinnaeve D., Rozenski J., Martins J.C., De Mot R. (2012). Genetic and functional characterization of cyclic lipopeptide white-line-inducing principle (WLIP) production by rice rhizosphere isolate *Pseudomonas putida* RW10S2. Appl. Environ. Microbiol..

[25] Omoboye O.O., Oni F.E., Batool H., Yimer H.Z., De Mot R., Hofte M. (2019). *Pseudomonas* cyclic lipopeptides suppress the rice blast fungus *Magnaporthe oryzae* by induced resistance and direct antagonism. Front. Plant. Sci..

[26] Vlassak K., Vanholm L., Duchateau L., Vanderleyden J., Demot R. (1992). Isolation and characterization of fluorescent *Pseudomonas* associated with the roots of rice and banana grown in Sri-lanka. Plant. Soil.

[27] De Souza J.T., Mazzola M., Raaijmakers J.M. (2003). Conservation of the response regulator gene gacA in *Pseudomonas* species. Environ. Microbiol..

[28] Mehnaz S., Saleem R.S.Z., Yameen B., Pianet I., Schnakenburg G., Pietraszkiewicz H., Valeriote F., Josten M., Sahl H.G., Franzblau S.G. (2013). Lahorenoic acids a-c, ortho-dialkyl-substituted aromatic acids from the biocontrol strain *Pseudomonas aurantiaca* PB-St2. J. Nat. Prod..

[29] Martin C., Ibanez R., Nothias L.F., Boya C.A., Reinert L.K., Rollins-Smithv L.A., Dorrestein P.C., Gutierrez M. (2019). Viscosin-like lipopeptides from frog skin bacteria inhibit *Aspergillus fumigatus* and *Batrachochytrium dendrobatidis* detected by imaging mass spectrometry and molecular networking. Sci. Rep..

[30] Geudens N., Nasir M.N., Crowet J.M., Raaijmakers J.M., Feher K., Coenye T., Martins J.C., Lins L., Sinnaeve D., Deleu M. (2017). Membrane interactions of natural cyclic lipodepsipeptides of the viscosin group. BBA-Biomembranes.

[31] De Vleeschouwer M., Van Kersavond T., Verleysen Y., Sinnaeve D., Coenye T., Martins J.C., Madder A. (2020). Identification of the molecular determinants involved in antimicrobial activity of pseudodesmin a, a cyclic lipopeptide from the viscosin group. Front. Microbiol..

[32] Thrane C., Olsson S., Nielsen T.H., Sorensen J. (1999). Vital fluorescent stains for detection of stress in *Pythium ultimum* and *Rhizoctonia solani* challenged with viscosinamide from *Pseudomonas fluorescens* DR54. FEMS Microbiol. Ecol..

[33] Olorunleke F.E. (2017). Cyclic Lipopeptides Produced by *Pseudomonas* spp. Associated with the Cocoyam (*Xanthosoma sagittifolium* (l.) Schott) Rhizosphere: Diversity, Regulation, Secretion and Biological Activity. Ph.D. Thesis.

[34] Omoboye O.O. (2019). Cyclic lipopeptide Diversity and Biocontrol Versatility of *Pseudomonas* species Associated with the Cocoyam Rhizosphere. Ph.D. Thesis.

[35] Gamborg O.L., Miller R.A., Ojima K. (1968). Nutrient requirements of suspension cultures of soybean root cells. Exp. Cell Res..

[36] Tambong J.T., Sapra V.T., Garton S. (1998). In vitro induction of tetraploids in colchicine-treated cocoyam plantlets. Euphytica.

[37] King E.O., Ward M.K., Raney D.E. (1954). Two simple media for the demonstration of pyocyanin and fluorescin. J. Lab. Clin. Med..

[38] Nielsen T.H., Sorensen D., Tobiasen C., Andersen J.B., Christophersen C., Givskov M., Sorensen J. (2002). Antibiotic and biosurfactant properties of cyclic lipopeptides produced by fluorescent *Pseudomonas* spp. From the sugar beet rhizosphere. Appl. Environ. Microbiol..

[39] Blin K., Shaw S., Steinke K., Villebro R., Ziemert N., Lee S.Y., Medema M.H., Weber T. (2019). antiSMASH 5.0: Updates to the secondary metabolite genome mining pipeline. Nucleic Acids Res..

[40] Rottig M., Medema M.H., Blin K., Weber T., Rausch C., Kohlbacher O. (2011). NRPSpredictor2-a web server for predicting NRPS adenylation domain specificity. Nucleic Acids Res..

[41] D’aes J., Kieu N.P., Leclere V., Tokarski C., Olorunleke F.E., De Maeyer K., Jacques P., Hofte M., Ongena M. (2014). To settle or to move? The interplay between two classes of cyclic lipopeptides in the biocontrol strain *Pseudomonas* CMR12a. Environ. Microbiol..

[42] Perneel M., Tambong J.T., Adiobo A., Floren C., Saborio F., Levesque A., Hofte M. (2006). Intraspecific variability of *Pythium myriotylum* isolated from cocoyam and other host crops. Mycol. Res..

[43] Nerey Y., Pannecoucque J., Hernandez H.P., Diaz M., Espinosa R., De Vos S., Van Beneden S., Herrera L., Hofte M. (2010). Rhizoctonia spp. causing root and hypocotyl rot in *Phaseolus vulgaris* in cuba. J. Phytopathol..

[44] Olorunleke F.E., Hua G.K.H., Kieu N.P., Ma Z.W., Hofte M. (2015). Interplay between orfamides, sessilins and phenazines in the control of Rhizoctonia diseases by *Pseudomonas* sp. CMR12a. Environ. Microbiol. Rep..

[45] Balibar C.J., Vaillancourt F.H., Walsh C.T. (2005). Generation of d amino acid residues in assembly of arthrofactin by dual condensation/epimerization domains. Chem. Biol..

[46] De Bruijn I., Raaijmakers J.M. (2009). Regulation of cyclic lipopeptide biosynthesis in *Pseudomonas fluorescens* by the Clpp protease. J. Bacteriol..

[47] Rokni-Zadeh H., Li W., Yilma E., Sanchez-Rodriguez A., De Mot R. (2013). Distinct lipopeptide production systems for WLIP (white line-inducing principle) in *Pseudomonas fluorescens* and *Pseudomonas putida*. Environ. Microbiol. Rep..

[48] Sajben E., Manczinger L., Nagy A., Kredics L., Vagvolgyi C. (2011). Characterization of pseudomonads isolated from decaying sporocarps of oyster mushroom. Microbiol. Res..

[49] Lalucat J., Mulet M., Gomila M., Garcia-Valdes E. (2020). Genomics in bacterial taxonomy: Impact on the genus *Pseudomonas*. Genes.

[50] Biessy A., Novinscak A., Blom J., Leger G., Thomashow L.S., Cazorla F.M., Josic D., Filion M. (2019). Diversity of phytobeneficial traits revealed by whole-genome analysis of worldwide-isolated phenazine-producing *Pseudomonas* spp. Environ. Microbiol..

[51] Broekaert W.F., Terras F.R.G., Cammue B.P.A., Osborn R.W. (1995). Plant defensins-novel antimicrobial peptides as components of the host-defense system. Plant. Physiol..

[52] Thomma B., Cammue B.P.A., Thevissen K. (2002). Plant defensins. Planta.

[53] Spelbrink R.G., Dilmac N., Allen A., Smith T.J., Shah D.M., Hockerman G.H. (2004). Differential antifungal and calcium channel-blocking activity among structurally related plant defensins. Plant. Physiol..

[54] Pombeiro-Sponchiado S.R., Sousa G.S., Andrade J.C.R., Lisboa H.F., Goncalves R.C.R. (2017). Production of melanin pigment by fungi and its biotechnological applications. Melanin.

[55] Ipcho S., Sundelin T., Erbs G., Kistler H.C., Newman M.A., Olsson S. (2016). Fungal innate immunity induced by bacterial microbe-associated molecular patterns (MAMPs). G3 (Bethesda).

[56] Adiobo A., Oumar O., Perneel M., Zok S., Hofte M. (2007). Variation of Pythium-induced cocoyam root rot severity in response to soil type. Soil Biol. Biochem..

[57] Schroeder K.L., Martin F.N., de Cock A., Levesque C.A., Spies C.F.J., Okubara P.A., Paulitz T.C. (2013). Molecular detection and quantification of Pythium species: Evolving taxonomy, new tools, and challenges. Plant. Dis..

[58] Hansen M., Thrane C., Olsson S., Sorensen J. (2000). Confocal imaging of living fungal hyphae challenged with the fungal antagonist viscosinamide. Mycologia.

[59] Dirksen P., Marsh S.A., Braker I., Heitland N., Wagner S., Nakad R., Mader S., Petersen C., Kowallik V., Rosenstiel P. (2016). The native microbiome of the nematode *Caenorhabditis elegans*: Gateway to a new host-microbiome model. BMC Biol..

[60] Silby M.W., Cerdeno-Tarraga A.M., Vernikos G.S., Giddens S.R., Jackson R.W., Preston G.M., Zhang X.X., Moon C.D., Gehrig S.M., Godfrey S.A. (2009). Genomic and genetic analyses of diversity and plant interactions of *Pseudomonas fluorescens*. Genome Biol..

