# Supplementary material for: Biosynthesis and Antimicrobial Activity of Pseudodesmin and Viscosinamide Cyclic Lipopeptides Produced by Pseudomonads Associated with the Cocoyam Rhizosphere
Source: Microorganisms. 2020 Jul 20;8(7):1079. doi: 10.3390/microorganisms8071079 (PMC7409209; doi:10.3390/microorganisms8071079)
Supplement: Supplementary file 1 [file microorganisms-08-01079-s001.pdf]

Supplementary materials

Biosynthesis and antimicrobial activity of pseudodesmin and viscosinamide cyclic lipopeptides produced by pseudomonads associated with the cocoyam rhizosphere

Feyisara E. Oni [1,2], Niels Geudens [3], Amayana Adiobo [4], Olumide O. Omoboye [1,5], Elsie A. Enow [1], Joseph T. Onyeka [6], Ayodeji E. Salami [7], René De Mot [8], José C. Martins [3], and Monica Höfte [1,*]

1 Laboratory of Phytopathology, Department of Plants and Crops, Faculty of Bioscience Engineering, Ghent University, Coupure Links 653, B-9000 Ghent, Belgium; feyisaraeyiwumi.olorunleke@ugent.be (F.E.O); oomoboye@oauife.edu.ng (O.O.O); ayamelsie@yahoo.com (E.A.E)
2 Unit for Environmental Sciences and Management, Faculty of Natural and Agricultural Sciences, North-West University, Potchefstroom, South Africa
3 NMR and Structure Analysis Unit, Department of Organic and Macromolecular Chemistry, Faculty of Science, Ghent University, Krijgslaan 281, B-9000 Gent, Belgium; niels.geudens@ugent.be (N.G.); jose.martins@ugent.be (J.C.M.)
4 Jay PJ Biotechnology Laboratory, Institute of Agricultural Research for Development (IRAD), Ekona, Cameroon; amayanadio@yahoo.com (A.A)
5 Department of Microbiology, Obafemi Awolowo University, Ile-Ife 220005, Osun State, Nigeria
6 Plant Pathology Unit, National Root Crops Research Institute (NRCRI) Umudike, Abia State, Nigeria; jonyeka@yahoo.com (J.T.O)
7 Faculty of Agriculture, Department of Crop, Horticulture and Landscape Design, Ekiti State University (EKSU), Ado-Ekiti, Nigeria; ayodeji.salami@eksu.edu.ng (A.E.A)
8 Centre of Microbial and Plant Genetics, Faculty of Bioscience Engineering, KU Leuven, Heverlee, Belgium; rene.demot@kuleuven.be (R.D.M)
* Correspondence: monica.hofte@ugent.be; Tel.: +32-9-264-6017

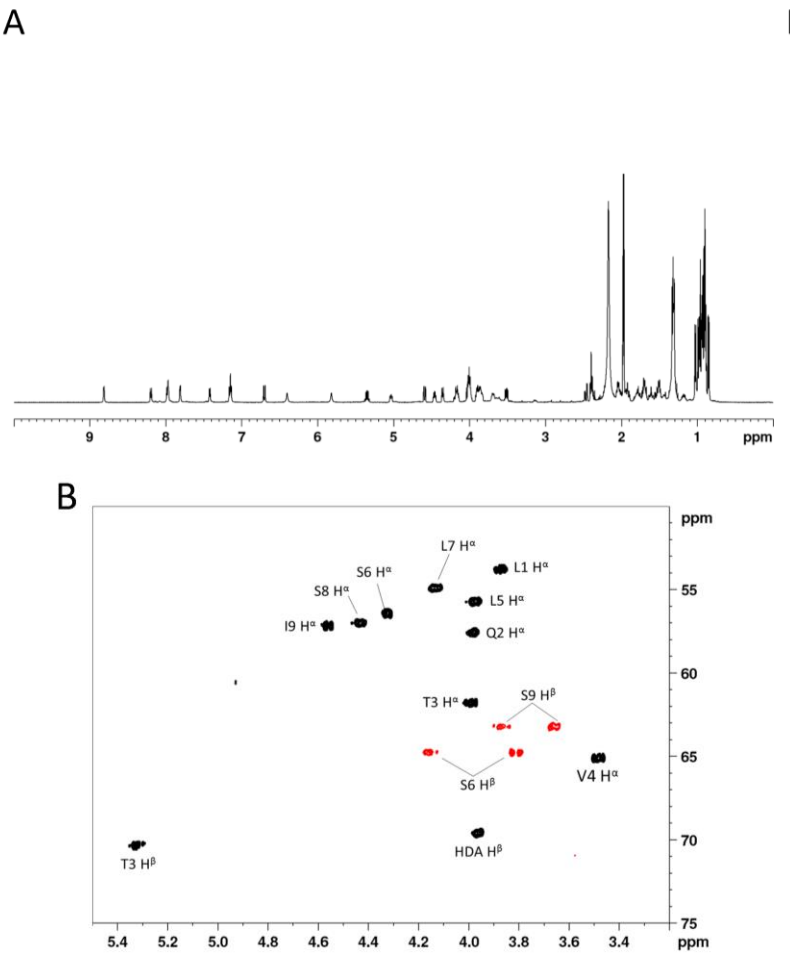

Figure S1. A) 1D ^{1}H NMR spectrum of major isolated CLP (N6, pseudodesmin A) (500 MHz, CD_3CN, 298K); **B)** The alpha region of a ^{1}H-^{13}C gHSQC spectrum of the isolated CLP shows the presence of 9 amino acids. The high chemical shift of the Thr3 CH$^{\beta}$ indicates that the C-terminal ester bond is formed with this residue.

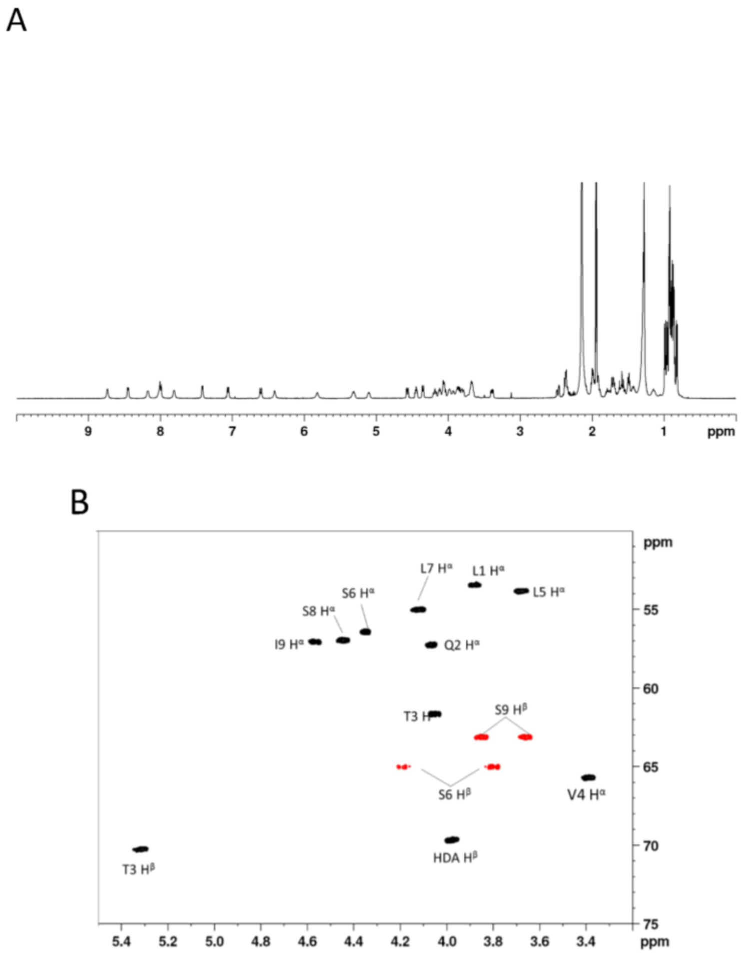

Figure S2. A) 1D ^{1}H NMR spectrum of viscosinamide A as extracted from A2W4.9 (500 MHz, CD$_3$CN, 298K); **B)** The alpha region of a ^{1}H-^{13}C gHSQC spectrum of the isolated CLP shows the presence of 9 amino acids. The high chemical shift of the Thr3 CH$^{\beta}$ indicates that the C-terminal ester bond is formed with this residue.

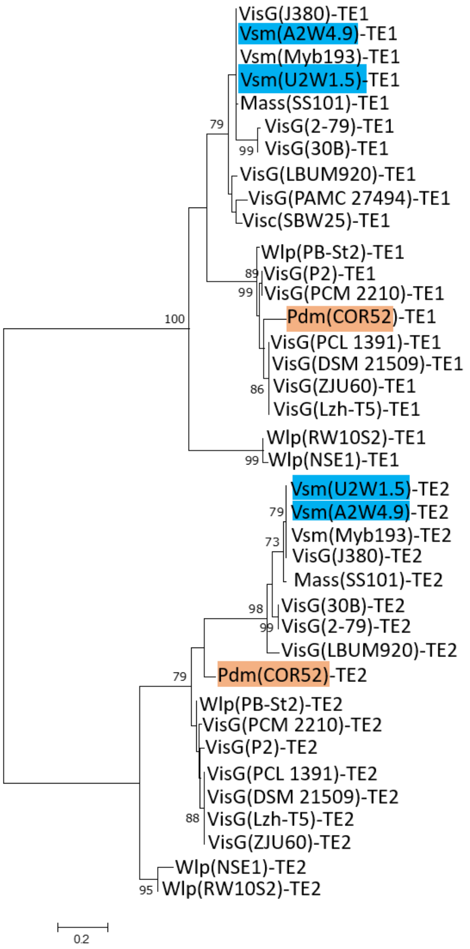

Figure S3. Phylogenetic analyses of the TE domains extracted from the modules of CLP gene clusters. Phylogenetic analyses of the Thioesterase (Te) domains extracted from functionally characterized and select putative *Pseudomonas* NRPSs. Lipopeptide-specific codes used for NRPS enzymes: Pdm (pseudodesmin, *Pseudomonas* sp. COR52, highlighted in blue); Vsm (viscosinamide, *Pseudomonas* sp. A2W4.9 and *Pseudomonas* sp. U2W1.5, highlighted in orange); Wlp (white line inducing principle, *Pseudomonas* sp. NSE1, *P. putida* RW10S2, *P. chlororaphis* Pb-St2); Mass (massetolide, *P. lactis* SS101); Visc (viscosin, *P. fluorescens* SBW25); VisG (viscosin group, not characterized: *Pseudomonas* sp. Myb193, *Pseudomonas* sp. J380, *P. synxantha* 30B, *P. synxantha* 2-79, *P. antarctica* PAMC 27494, *Pseudomonas* sp. LBUM920, *P. chlororaphis* subsp. *piscium* PCL1391, *P. chlororaphis* subsp. *aurantiaca* PCM 2210, *P. chlororaphis* subsp. *aureofaciens* P2, *P. chlororaphis* subsp. *piscium* DSM 21509, *P. chlororaphis* subsp. *piscium* ZJU60 and *P. chlororaphis* Lzh-T5). For each domain, the substrate specificity is indicated in parentheses, using the standard amino acid three-letter code. VisG represent NRPS enzymes for Viscosin Group CLPs that have been characterised based on genome mining only. Phylogenetic tree was drawn using the Geneious 11.1.5 software.

Table S1. ^1H and ^{13}C chemical shift values of the isolated compound (N6, pseudodesmin A) (500 MHz, CD_3CN, 298K).

			^1H δ	^{13}C δ				^1H δ	^{13}C δ
**HDA**					**Leu5**				
		CO	-	175.61	$^3J_{HNH\alpha}$	n.d.	NH	7.94	-
		$CH_2\alpha1$	2.44	44.75			$CH\alpha$	3.97	55.68
		$CH_2\alpha2$	2.35	44.75			CO	-	173.77
		$CH\beta$	3.96	69.58			$CH_2\beta1$	1.67	40.63
		$CH_2\gamma$	1.48	38.23			$CH_2\beta2$	1.51	40.63
		$CH_2\delta1$	1.44	26.31			$CH\gamma$	1.77	25.49
		$CH_2\delta2$	1.30	26.31			$CH_3\delta1$	0.88	21.29
		$CH_2\varepsilon$	1.30	30.11			$CH_3\delta2$	0.86	23.33
		$CH_2\zeta$	1.28	30.29					
		$CH_2\eta$	1.28	32.62	**Ser6**				
		$CH_2\theta$	1.29	23.39	$^3J_{HNH\alpha}$	n.d.	NH	7.12	-
		$CH_2\iota$	0.88	14.46			$CH\alpha$	4.33	56.40
		OH	n.d.	-			CO	-	172.26
							$CH_2\beta1$	4.15	64.72
**Leu1**							$CH_2\beta2$	3.81	64.72
$^3J_{HNH\alpha}$	4.1 Hz	NH	7.78	-			OH	5.00	-
		$CH\alpha$	3.87	53.70					
		CO	-	175.64	**Leu7**				
		$CH_2\beta1$	1.75	39.29	$^3J_{HNH\alpha}$	n.d.	NH	7.11	-
		$CH_2\beta2$	1.66	39.29			$CH\alpha$	4.13	54.87
		$CH\gamma$	1.67	25.42			CO	-	174.31
		$CH_3\delta1$	0.94	23.22			$CH_2\beta1$	1.89	42.06
		$CH_3\delta2$	0.90	22.08			$CH_2\beta2$	1.58	42.06
							$CH\gamma$	1.90	25.51
**Gln2**							$CH_3\delta1$	0.99	23.47
$^3J_{HNH\alpha}$	3.9 Hz	NH	8.78	-			$CH_3\delta2$	0.90	21.35
		$CH\alpha$	3.98	57.54					
		CO	-	177.19	**Ser8**				
		$CH_2\beta$	2.02	26.42	$^3J_{HNH\alpha}$	n.d.	NH	7.50	-
		$CH_2\gamma$	2.37	31.96			$CH\alpha$	4.43	56.97
		$CO\delta$	-	176.33			CO	-	172.36
		OH	n.d.	-			$CH_2\beta1$	3.87	63.17
							$CH_2\beta2$	3.66	63.17
**Thr3**							OH	3.81	-
$^3J_{HNH\alpha}$	7.2 Hz	NH	8.16	-					
		$CH\alpha$	3.99	61.76	**Ile9**				
		CO	-	174.47	$^3J_{HNH\alpha}$	10.2 Hz	NH	6.67	-
		$CH_2\beta$	5.32	70.32			$CH\alpha$	4.56	57.16
		$CH\gamma$	1.30	18.50			CO	-	170.38
							$CH\beta$	1.97	36.93
**Val4**							$CH_2\gamma1$	1.16	25.22
$^3J_{HNH\alpha}$	6.2 Hz	NH	7.39	-			$CH_2\gamma2$	0.97	25.22
		$CH\alpha$	3.48	65.09			$CH_3\gamma$	0.82	16.24
		CO	-	174.78			$CH_3\delta2$	0.86	12.29
		$CH\beta$	2.18	29.89					
		$CH_1\gamma1$	0.95	21.08					
		$CH_2\gamma2$	0.92	19.50					

n.d: not determined.

Table S2. ^{1}H and ^{13}C chemical shift values of viscosinamide A as extracted from *P.* sp. A2W4.9. (500 MHz, CD_3CN, 298K).

			^{1}H δ	^{13}C δ
**HDA**				
		CO	-	175.34
		CH_2α1	2.47	44.73
		CH_2α2	2.36	44.73
		CHβ	3.98	69.66
		CH_2γ	1.49	38.10
		CH_2δ1	1.43	26.36
		CH_2δ2	1.31	26.36
		CH_2ε	1.31	30.02
		CH_2ζ	1.31	30.02
		CH_2η	1.28	32.60
		CH_2θ	1.29	32.30
		CH_2ι	0.88	14.33
		OH	n.d.	-
**Leu1**				
$^3J_{HNH\alpha}$	n.d.	NH	7.99	-
		CHα	3.87	53.41
		CO	-	n.d.
		CH_2β1	1.79	39.14
		CH_2β2	1.71	39.14
		CHγ	1.69	25.27
		CH_3δ1	0.92	23.21
		CH_3δ2	0.89	21.89
**Gln2**				
$^3J_{HNH\alpha}$	n.d.	NH	8.73	-
		CHα	4.07	57.24
		CO	-	n.d.
		CH_2β	1.99	26.39
		CH_2γ	2.36	32.00
		COδ	-	n.d.
		NH_2	6.41/5.81	-
**Thr3**				
$^3J_{HNH\alpha}$	n.d.	NH	8.17	-
		CHα	4.05	61.65
		CO	-	n.d.
		CH_2β	5.32	70.27
		CHγ	1.28	18.38
**Val4**				
$^3J_{HNH\alpha}$	n.d.	NH	7.81	-
		CHα	3.39	65.69
		CO	-	174.00
		CHβ	2.11	29.98
		CH_1γ1	0.95	20.76
		CH_2γ2	0.92	19.48
**Leu5**				
$^3J_{HNH\alpha}$	6.3 Hz	NH	8.45	-
		CHα	3.68	53.81
		CO	-	n.d.
		CH_2β1	1.94	37.35
		CH_2β2	1.73	37.35
		CHγ	1.61	25.50
		CH_3δ1	0.83	23.92
		CH_3δ2	0.86	21.28
**Ser6**				
$^3J_{HNH\alpha}$	8.1 Hz	NH	7.06	-
		CHα	4.35	56.42
		CO	-	172.30
		CH_2β1	4.18	65.03
		CH_2β2	3.80	65.03
		OH	5.10	-
**Leu7**				
$^3J_{HNH\alpha}$	5.6 Hz	NH	7.41	-
		CHα	4.12	54.99
		CO	-	n.d.
		CH_2β1	1.91	41.98
		CH_2β2	1.55	41.91
		CHγ	1.93	35.35
		CH_3δ1	0.98	23.31
		CH_3δ2	0.91	21.41
**Ser8**				
$^3J_{HNH\alpha}$	n.d.	NH	8.00	-
		CHα	4.45	56.94
		CO	-	n.d.
		CH_2β1	3.85	63.12
		CH_2β2	3.66	63.12
		OH	3.92	-
**Ile9**				
$^3J_{HNH\alpha}$	10.0 Hz	NH	6.60	-
		CHα	4.56	57.04
		CO	-	n.d.
		CHβ	1.99	36.75
		CH_2γ1	1.15	25.11
		CH_2γ2	0.95	25.11
		CH_3γ	0.82	16.13
		CH_3δ2	0.86	12.21

n.d: not determined.
